# Particulate matter 2.5 promotes bladder cancer cell migration and invasion through the crosstalk between integrin-mediated MAPK/ERK and Wnt/β-catenin pathways

**DOI:** 10.1186/s12989-025-00656-3

**Published:** 2026-01-16

**Authors:** Yung-Ting Cheng, Kai-Hsi Lu, Shu-Ying Hong, Chung-Hsin Chen, Chao-Yuan Huang, Hsiu-Ni Kung

**Affiliations:** 1 Department of Urology, National Taiwan University Hospital Hsin-Chu Branch BioMedical Park Hospital, No. 2, Sec. 1, Shengyi Rd., 302058 Zhubei City, Hsin-Chu County, Taiwan; 2 Graduate Institute of Anatomy and Cell Biology, College of Medicine, National Taiwan University, 6F, No.1, Sec. 1, Jen-Ai Road, Taipei City, 100233 Taiwan; 3 Telehealth and International Medical Services Center, Cheng-Hsin General Hospital, Taipei City, 112401 Taiwan; 4 Department of Urology, National Taiwan University Hospital, Taipei City, 100225 Taiwan

**Keywords:** PM_2.5_, Bladder cancer, Cell migration, Cell invasion, Wnt/β-catenin pathway, MAPK/ERK pathway

## Abstract

**Background:**

Fine particulate matter 2.5 (PM_2.5_), a key indicator of air pollution, is classified as a human carcinogen. However, the link between air pollution and bladder cancer (BC) progression remains unclear. Dysregulation of the Wingless-related integration site (Wnt)/β-catenin and mitogen-activated protein kinase (MAPK)/extracellular signal-regulated kinase (ERK) pathways is a key driver of tumorigenesis in multiple cancers, including BC.

**Results:**

This study demonstrated that PM_2.5_ exposure enhances BC cell migration and invasion. Ribonucleic acid (RNA) sequencing identified the Wnt signaling pathway as a key regulator in PM_2.5_-exposed BC cells. Elevated protein levels of Wnt3A, Wnt5A, and β-catenin, along with the nuclear translocation of β-catenin, further highlighted the role of the PM_2.5_-activated Wnt/β-catenin pathway in promoting BC progression. The interaction between the Wnt/β-catenin and MAPK/ ERK pathways was examined using inhibitors and shRNAs. MEK or ERK inhibition not only suppressed PM_2.5_-induced upregulation of Wnt3A, Wnt5A, and β-catenin nuclear translocation but also significantly reduced the migration and invasion of PM_2.5_-exposed BC cells. Both pathways represent promising therapeutic targets, and several existing pathway-specific inhibitors may be repurposed for the future clinical management of PM_2.5_-induced BC progression.

**Conclusions:**

PM_2.5_ promotes BC progression through both the MAPK/ERK and Wnt/β-catenin signaling pathways. MEK/ERK inhibition suppressed PM_2.5_-induced nuclear translocation of β-catenin, suggesting that the MAPK/ERK pathway functions upstream of the Wnt/β-catenin pathway. This study provides mechanistic insights into how PM_2.5_ exposure drives BC progression and offers a potential foundation for developing targeted therapies for PM_2.5_-associated BC.

**Supplementary Information:**

The online version contains supplementary material available at 10.1186/s12989-025-00656-3.

## Background

The increasingly prevalent ambient air pollution has led to numerous health issues worldwide. Air pollution is the leading contributor to disability-adjusted life-years (DALYs) and the second most significant risk factor for mortality worldwide, with approximately 11% of its health burden attributable to cancer [1, 2]. The International Agency for Research on Cancer (IARC) of the WHO has classified air pollution as a Group 1 human carcinogen, based on strong evidence linking it to lung cancer and suggesting a positive association with bladder cancer (BC) [3, 4]. BC is the nineth most common cancer worldwide, with an estimated 200,000 deaths in 2022 [5]. Smoking is the primary risk factor for BC, followed by occupational exposure to aromatic amines or polycyclic aromatic hydrocarbons (PAHs), which accounts for 5.7% of cases [6]. The urinary system is exposed to carcinogenic metabolites from tobacco through urinary metabolism [7]. Regardless of tobacco use, carcinogenic metabolites from air pollutants have been detected in BC patients but not in non-BC individuals, suggesting potential environmental exposure effects on BC development [8, 9].

As the primary indicator of air pollution, fine particulate matter 2.5 (PM_2.5_) is defined by particles with a diameter of less than or equal to 2.5 μm, formed after photochemical reactions or fuel combustion. After being inhaled, particles carrying various organic substances, including PAHs or acrolein, circulate throughout the body via the bloodstream [10]. Apart from individual compounds, carcinogenicity can be triggered by multiple components involved in complex cellular responses [11, 12]. PM_2.5_-exposed lung epithelial and lung cancer cells exhibit increased proliferation through cell cycle regulation via the phosphatidylinositol 3-kinase (PI3K)/ Ak strain transforming (Akt, Protein Kinase B, PKB) and mitogen-activated protein kinase (MAPK) pathways. PM_2.5_ exposure also disrupts cell junctions and reorganizes the cytoskeleton, facilitating migration and invasion [13, 14].

The Spanish Bladder Cancer Study found that living for more than 40 years in cities with over 100,000 residents was significantly associated with a higher risk of BC [9]. A 16-year retrospective cohort study conducted in Taiwan similarly reported that long-term residence in areas with elevated PM_2.5_ levels was significantly associated with a higher risk of urothelial carcinoma [4]. Yeh et al. found PM_2.5_ concentrations were positively correlated with BC mortality among both genders in Taiwan [15]. However, some studies have shown inconsistent results [16]. Wang et al. reported that PM_2.5_ exposure was positively associated with BC incidence but inversely associated with BC mortality [17]. Zare Sakhvidi et al. reviewed eighteen studies on long-term air pollution exposure and BC risk, concluding that the evidence was more suggestive for mortality than for incidence [3]. In the present study, we hypothesize PM_2.5_ as promoter of existing BC pathology.

Activation of the Wnt/β-catenin pathway plays a pivotal role in cancer stem cell maintenance, tumor progression, metastasis, and therapeutic resistance [18–20]. The Wnt signaling pathway is classified into the canonical β-catenin-dependent and non-canonical β-catenin-independent pathways [18]. In canonical Wnt signaling, Wnt ligands interact with Frizzled (Fzd) and low-density lipoprotein receptor-related protein (LRP) 5/6 receptors, activating dishevelled (DVL), which inhibits the β-catenin destruction complex composed of adenomatous polyposis coli (APC), axis inhibition protein (AXIN), glycogen synthase kinase 3β (GSK-3β), and casein kinase 1 (CK1). Unphosphorylated β-catenin escapes proteasomal degradation, accumulates in the cytoplasm, and translocates into the nucleus, activating target gene transcription [18]. Dysregulation of the Wnt signaling pathway occurs in 25% of urothelial cell carcinomas [21]. Compared with normal bladder mucosa, BC exhibited a significantly higher frequency of nuclear β-catenin accumulation, which was inversely associated with Wnt inhibitory factor-1 expression [22]. Wnt5A expression in BC correlates with pathological stage and histological grade, promotes cell migration and invasion, and is associated with chemoresistance [18, 23, 24]. Aberrant β-catenin expression was significantly associated with reduced overall survival and disease-specific survival in BC [25, 26]. PM_2.5_ can induce epithelial–mesenchymal transition (EMT) through activation of the Wnt/β-catenin signaling pathway [27]. Xu et al. reported PM₂.₅-induced Wnt3A-enriched exosomes activated the Wnt/β-catenin signaling to promote lung alveolar cancer cell proliferation and xenograft tumor growth [28].

However, β-catenin activation alone does not induce BC [21, 29]. The Cancer Genome Atlas (TCGA) data reported that 68% of muscle-invasive BC patients possess genomic alterations potentially targetable by therapy, including recurrent receptor tyrosine kinase (RTK)–MAPK pathway alterations found in 45% of cases [30]. Dysregulated signaling between the Rat Sarcoma (RAS)-Rapidly Accelerated Fibrosarcoma(RAF)-MAPK kinase (MEK) and ERK pathway is associated with multiple human cancers [31]. Tobacco exposure has been reported to enhance BC progression via extracellular signal-regulated kinases 1 and 2 (ERK1/2) pathway [32]. ERK1/2 is a subfamily of the MAPK pathway, which is a key signaling cascade that regulates various cellular processes and also includes p38 kinases and c-Jun N-terminal kinases (JNKs) subfamilies [32]. The interactions between Wnt/β-catenin and MAPK/ERK pathways have been linked to cancer progression [33]. Wnt3A protein is reported to directly stimulate the MAPK pathway [34]. Wnt signaling is also shown to prevent Ras proteins in the MAPK pathway from being phosphorylated by GSK-3β, thereby preventing their proteasomal degradation [33]. The synergistic interaction is evidenced by the co-occurrence of KRas and APC mutations in colorectal tumorigenesis [33]. Even in the absence of a KRas mutation, increased β-catenin and RAS staining in colorectal cancer (CRC) patients with APC mutations provides further evidence of Ras stabilization by the Wnt/β-catenin pathway [35]. Such cooperation between pathways in driving BC formation has also been demonstrated in mice [21].

To the best of our knowledge, no in vitro studies have explored the mechanisms of PM_2.5_-induced bladder carcinogenesis. This study aims to investigate how PM_2.5_ affects the migration and invasion abilities of BC cells, providing novel insights into PM_2.5_-induced bladder oncogenesis and potential therapeutic targets.

## Methods

### Clinical statistics of PM_2.5_ exposure and BC risk

Public statistics on PM_2.5_ levels were retrieved from the websites of the Environmental Protection Administration, Executive Yuan, and data on age-adjusted incidence and death rates were obtained from the Cancer Registry Database established by the Ministry of Health and Welfare and the National Health Insurance Administration. Taiwan’s Ministry of Environment uses the NIEA A205.11 C method to measure PM_2.5_ in the air. This manual method collects fine particles (≤ 2.5 μm) over 24 h using a special sampler and filter. The filter is weighed before and after sampling under controlled conditions. By dividing the net particle mass by the total air volume sampled, the 24-hour PM_2.5_ concentration is calculated. Individuals diagnosed with BC or those eligible for BC-related deaths were identified according to the International Classification of Diseases, 9th and 10th Revisions (ICD-9 and ICD-10), codes 188 and C67, respectively. This study was approved by the Research Ethics Committee Office of National Taiwan University Hospital (202006045  W), with a waiver of informed consent due to its retrospective nature. The annual concentration of PM_2.5_ (µg/m³) for 20 counties and cities in Taiwan (excluding Kinmen County and Lienchiang County) was averaged from 2013 to 2019. Age-standardized incidence and mortality rates (ASIR or ASMR) of BC were calculated using the year 2000 world standard population, applying the following formula:

$$\:ASIR\:or\:ASMR={\sum\:}_{i}\left(\raisebox{1ex}{${n}_{i}$}\!\left/\:\!\raisebox{-1ex}{${N}_{i}\text{}$}\right.\times\:{w}_{i}\right)$$

- $\:{n}_{i}$ = number of cases (or deaths) in age group *i*.
- $\:{N}_{i}$ = population size in age group *i*.
- $$\:\raisebox{1ex}{${n}_{i}$}\!\left/\:\!\raisebox{-1ex}{${N}_{i}\text{}$}\right.$$= age-specific rate in group *i*.
- $\:{w}_{i}$ = standard population weight for age group *i* (per 100,000).

The results aim to provide an observational analysis of local BC incidence and mortality rates in relation to air pollution, serving as a clinical reference for future experimental hypotheses.

### PM_2.5_ preparation

PM_2.5_ was purchased from National Institute of Standards and Technology (NIST, USA), encoded as Standard Reference Material (SRM) 2786. This SRM was collected from Prague, Czech Republic in 2005, representing general atmospheric PM from an urban area. Particles collected on Teflon filters were resuspended using a particle suspension unit and an ultra-high-volume sampler (UHVS). Certified mass fraction values for PAH, inorganic constituents, and metals in the PM were provided in detail. Each unit of SRM2786 contains 100 to 140 mg of PM. The samples were diluted to a final concentration of 1 mg/ml with phosphate buffered saline (PBS, GeneTex, California, USA, GTX48887), aliquoted, and stored at -20 °C. Before being added to cells, PM_2.5_ was thawed at room temperature. Uniform dispersion of the particles was ensured by vortexing or gently pipetting of the solution.

### Cell culture and treatment

The human bladder transitional cell carcinoma cell line T24 and the Tri-Service General Hospital 8301 (TSGH 8301) were purchased from the Bioresource Collection and Research Center (BCRC, Hsinchu, Taiwan). Both cell lines were cultured in Dulbecco’s Modified Eagle Medium (DMEM, Gibco, Thermo Fisher Scientific, New Taipei City, Taiwan, 2383696) supplemented with 1% of Penicillin-Streptomycin (Gibco, Thermo Fisher Scientific, 585609) and 10% Fetal Bovine Serum (FBS, Gibco, Thermo Fisher Scientific, 2389940RP), and incubated at 37 °C under 5% CO_2_. Cells were maintained in 10-cm dishes and passaged upon reaching 70–80% confluence. After removing the culture medium, cells were rinsed with PBS and treated with 0.1% Trypsin-Ethylenediaminetetraacetic acid (EDTA; Gibco, Thermo Fisher Scientific, 2381444) at 37 °C for 3 min. Once detachment was confirmed, the reaction was quenched by adding FBS. The cells were then centrifuged at 1000 rpm for 3 min to remove the supernatant. Cell pellets were gently resuspended in 1 ml of culture medium. To assess cell viability and count, a mixture of 10 µL of cell suspension and 0.4% trypan blue (Logos Biosystems, Gyeonggi-do, South Korea, T13001) was prepared and loaded onto an automated cell counter (LUNA-II™, Logos Biosystems).

### Wound healing assay

Two well culture-inserts (Ibidi, Gräfelfing, Germany, IB-80209) placed at the center of 24-well plates were used to create uniform wounds. Cells were seeded into each insert with 70 µL of cell suspension and 200 µL of culture medium added outside the insert. Once the cells reached confluence within the inserts, both the inserts and the culture medium were gently removed. After treatment with PM_2.5_, the distance between the edges of the wound margins was photographed under a microscope at specified time intervals. The migrated distance was then calculated using ImageJ software (https://imagej.nih.gov/ij/). Two to three fields were imaged per well, and the averaged value was considered as one data point (“n”). Four replicates (*n* = 4) were performed in each experiment as technical replicates to ensure consistency under the same experimental conditions, and the experiment was independently repeated twice as biological replicates.

### Transwell migration and invasion assays

24-well transwell chambers with 8.0 μm pore (JET Biofil, Guangzhou, China, TCS003024) were used to perform both transwell migration and invasion assays. T24 cells (2 × 10^4^ cells/100 µL) and TSGH 8301 cells (5 × 10^4^ cells/100 µL) suspended in serum-free culture medium were seeded into the upper chambers. For invasion assays, the upper chambers were pre-coated with 20 µL of 1 mg/mL Matrigel (Corning, New York, USA, 354230). A total of 500 µl of DMEM containing serum was added to the lower chamber. The cells were exposed to PM_2.5_ and incubated for 48 h at 37 °C. After incubation, migrated or invaded cells were fixed with 4% paraformaldehyde (PFA; Panreac AppliChem, Monza, Italy, 141451.1211) overnight. The following day, cells were stained with crystal violet for 3 min and then washed with double-distilled water (ddH₂O). Non-migrated/non-invaded cells remaining in the upper chamber were gently removed using cotton swabs. Stained cells on the lower side of the membrane were imaged in four random fields using a FLoid™ Cell Imaging Station (Thermo Fisher Scientific, Waltham, USA). The number of cells in each field was quantified using ImageJ, and the average cell count from the four fields was considered as one data point (“n”). Three technical replicates were performed, and the entire experiment was repeated two to three times as biological replicates.

### RNA isolation

RNA was isolated from TSGH 8301 cells, including two control groups and two PM_2.5_-treated groups, following exposure to 1 µg/mL PM_2.5_ for 6 h. After exposure, the culture medium was removed, and 800 µL of Trizol (EBL Biotechnology, Taipei, Taiwan, MRE-3200) was added to each well. The lysates were transferred to Eppendorf tubes containing 80 µL of 1-Bromo-3-chloro-propane (BCP, Sigma-Aldrich, B9673-200ML). The samples were mixed by vortexing for 15 s and centrifuged at 12,000 rpm for 15 min to separate the aqueous phase. The aqueous phase was transferred to a new Eppendorf tube containing an equal volume of isopropanol (Kanto Chemical, Tokyo, Japan, 32435-00). After a 10-minute incubation, the mixture was centrifuged at 12,000 rpm for 5 min to precipitate the RNA. The supernatant was discarded, and 1 mL of 75% ethanol/Diethyl Pyrocarbonate (DEPC; Sigma-Aldrich, 053K3652)-treated water was added for washing. The sample was centrifuged twice at 12,000 rpm for 5 min each to further purify the RNA. Following the removal of the supernatant and evaporation of residual ethanol, RNA was dissolved in 20 µL of DEPC-treated water on a preheated hotplate at 55 °C for 10 min. The concentration and purity of RNA were measured using a NanoDrop 2000 spectrophotometer (Thermo Fisher Scientific). A260/280 and A260/230 ratios close to 2 confirmed the absence of contaminants in the RNA samples.

### RNA sequencing and bioinformatics

RNA sequencing (RNA-seq) was performed by AZENTA life sciences (Massachusetts, USA). Briefly, 1 µg total RNA was used for library preparation. Poly(A)mRNA was isolated using Oligo(dT) beads and fragmented. First- and second-strand complementary deoxyribonucleic acid (cDNA) were synthesized using random primers. The purified double-stranded cDNA underwent end repair and dA-tailing in a single reaction, followed by T-A ligation to attach adaptors to both ends. The adaptor-ligated DNA was then size-selected using DNA clean beads. Subsequently, each sample underwent polymerase chain reaction (PCR) amplification using correlated primers, and the PCR products were verified. Libraries with different indices were multiplexed and loaded onto an Illumina HiSeq, Illumina Novaseq, or MGI2000 instrument for sequencing, following the manufacturer’s instructions, using a 2 × 150 bp paired-end configuration. RNA quality was assessed using Cutadapt (V1.9.1), and clean reads were mapped to the reference genome using Hisat2 (v2.0.1). HTSeq (v0.6.1) was employed to estimate gene and isoform expression levels from the paired-end clean data. Differential expression analysis was conducted using the DESeq2 Bioconductor package. Genes with an adjusted p-value (Padj) less than 0.05 were considered differentially expressed (DE). GOSeq (v1.34.1) was used to identify Gene Ontology (GO) terms with a significant Padj value (< 0.05). Additionally, the topGO package was utilized to generate a Directed Acyclic Graph (DAG), providing insights into the functional annotations of the enriched genes. KEGG (Kyoto Encyclopedia of Genes and Genomes) pathway enrichment analysis was performed to identify significant DE genes involved in biological pathways.

### Western blot analysis

The expressed proteins were extracted using a sample buffer containing 4.5 ml glycerol (Kanto Chemical, 17029-00), 0.355 g Tris (Bionovas, Toronto, Canada, AE0010), 0.9 g Sodium Dodecyl Sulphate (SDS, Bionovas, AS0070), 33.5 mg EDTA (Bionovas, AE0010), 0.75 ml 2-mercaptoethanol (Bionovas, AB0590), and 3.75 mg bromophenol blue (Millipore, Sigma-Aldrich, 108122). A 12% sodium dodecyl sulfate–polyacrylamide gel electrophoresis (SDS-PAGE) gel was prepared using 30% acrylamide (Bio-Rad, Taipei, Taiwan, 1610158), 0.375 M Tris-HCl (pH 8.8), 75% glycerol, 10% ammonium persulfate (APS; BioBasic, New York, USA AB0072), and tetramethylethylenediamine (TEMED; Bionovas, AT0170). The 4% stacking gel was prepared using 30% acrylamide, 0.375 M Tris-HCl (pH 6.8), 10% APS and TEMED. Equal amounts of protein samples were separated by electrophoresis at 120 V for 2 h and transferred onto nitrocellulose membrane (Pall Corporation, New York, USA, 66485) at 100 V for 150 min. Membranes were blocked with 5% bovine serum albumin (BSA; APOLO, Hsinchu County, Taiwan, APL-0017), followed by washing with Tris-buffered saline containing 0.1% Tween 20 (TBST; Emperor Chemical, Zhejiang, China 20130315023), and incubated with primary antibodies overnight at 4℃ (**Table 1**). After three washes with TBST (5 min each), the membranes were incubated with secondary antibodies at room temperature for 1 h. Protein signals were detected using enhanced chemiluminescent reagents (ECL, T-Pro Biotechnology, JT96-K004M) and captured with a UVP ChemStudio PLUS Touch (Analytik Jena, Jena, Germany) or Touch Imager (eBlot, Shanghai, China). The band intensities were quantified using Image J software and normalized to the internal control (GAPDH).

**Table 1:** Primary and secondary antibodies applied in Western blot analysis

Primary antibody	Species	kda	Titer	Manufacturer	Catalog
MMP1	Rabbit	54	1:1000	GeneTex	GTX100534
MMP14	Rabbit	66	1:1000	GeneTex	GTX132884
MMP2	Rabbit	74	1:1000	GeneTex	GTX636525
MMP9	Rabbit	78	1:1000	GeneTex	GTX100458
p-ERK1/2	Rabbit	44/42	1:1000	Chemicon	AB3826
ERK1/2	Rabbit	44/42	1:1000	Santa Cruz	sc-93/sc-154
Integrin β1	Rabbit	88	1:1000	GeneTex	GTX128839
GAPDH	Rabbit	36	1:1000	GeneTex	GTX100118
Wnt3A	Rabbit	39	1:1000	GeneTex	GTX128101
Wnt5A	Rabbit	42	1:1000	GeneTex	GTX111187
β-catenin	Mouse	92	1:1000	BD Biosciences	610,153
p(ser473)-AKT1	Rabbit	56	1:1000	GeneTex	GTX128414
AKT1	Mouse	62	1:100	Santa cruz	sc-5298

**Table:** 

Secondary antibody	Titer	Manufactor	Catalog
Peroxidase-AffiniPure Goat Anti-Rabbit IgG	1:5000	Jackson ImmunoResearch	111-035-003
Peroxidase-AffiniPure Goat Anti-Mouse IgG	1:5000	Jackson ImmunoResearch	115-035-003

### Immunocytochemistry (ICC)

T24 and TSGH 8301 cells treated with PM_2.5_ on coverslips were fixed with 4% PFA overnight. After fixation, cells were washed three times with PBS and permeabilized with 0.5% Triton X-100 (Sigma-Aldrich, X100-100ML) in 10% FBS/PBS at room temperature for 1 h. Cells were then washed again three times with PBS. For immunofluorescence staining, actin-phalloidin (1:200) was applied to the coverslips and incubated at room temperature for 1 h, protected from light. For ICC, cells were incubated with 60 µl of primary antibody against β-catenin (1:200, BD Biosciences, 610153) at 4℃ overnight. After three washes with PBS to remove excess primary antibodies, cells were incubated with 60 µl of fluorescent secondary antibody, Goat Anti-Mouse IgG Alexa Fluor^®^ 488 (1:400, Abcam, Cambridge, UK, ab150117), protected from light at room temperature for 1 h. Following another three PBS washes, 60 µl Hoechst (1:20,000 in PBS, Thermo Fisher Scientific, 62249) was added for 10 min to stain nuclei, followed by three additional PBS washes. Coverslips were mounted on glass slides with 50% glycerol/PBS. Images were acquired using a fluorescence microscope (Leica DM RE, TCS SP2) equipped with red (640 nm), green (520 nm), and blue (440 nm) filters. Nuclear translocation of β-catenin was analyzed using ImageJ software. Six random images were captured per slide, and each image was considered as one data point (“n”). Three independent experiments were performed to confirm the consistency of the results.

### Enzyme-linked immunosorbent assay (ELISA)

ELISA kits were used to measure Wnt3A (Fine Test, EH2146) and Wnt5A (BT LAB, E2352Hu). After seeding onto 24-well plates (TSGH8301: 7 × 10^4^ cells/well, T24: 5 × 10^4^ cells/well), cells were treated with PM_2.5_ (TSGH8301: 1.25 µg/ml, T24: 2.5 µg/ml) for 15 min, 30 min, and 24 h, respectively. Conditioned media were collected and centrifuged at 4℃ and 2500 rpm for 5 min for Wnt3A and 20 min for Wnt5A to obtain the supernatant. Standard curves were prepared using the provided strips (0–2000 pg/mL, 100 µL/well for Wnt3A; 0–16 ng/mL, 50 µL/well for Wnt5A). For samples, 100 µL of conditioned medium per well was added for Wnt3A, while 40 µl/well of conditioned medium and 10 µl of Wnt5A antibody were added for Wnt5A. 50 µl of streptavidin-HRP was added to both standards and samples for Wnt5A. Plates were sealed and incubated at 37℃ for 90 min (Wnt3A) and 60 min (Wnt5A). After thorough washing with the wash buffer, 100 µL/well of biotin-labeled antibody working solution, 100 µL/well of streptavidin-biotin complex (SABC) working solution, and 90 µL/well of 3,3’,5,5’-tetramethylbenzidine (TMB) substrate solution was sequentially added for Wnt3A detection, with each step followed by a wash. For Wnt5A detection, 50 µL/well of Substrate Solution A and 50 µL/well of Substrate Solution B were added. Finally, the plates were sealed and incubated at 37℃ for 10 min, followed by the addition of 50 µl/well of stop solution, protected from light. Optical density (OD) at 450 nm was measured immediately.

### Short hairpin RNA (shRNA) transfection

The endogenous MAPK pathway was knocked down using shRNA viral strains targeting the sequences CAGGTACCTGGAGTTTAATAC and CCCATATCTGGAGCAGTATTA. The shRNA viral strains (clone IDs: TRCN0000195472 and TRCN0000195517) were purchased from the RNA Technology Platform and Gene Manipulation Core (RNAi core) of the National Science and Technology Council in Taiwan. Based on the provided relative infection unit (RIU/µL), a multiplicity of infection (M.O.I) of 3 was used. T24 and TSGH 8301 cells were seeded into 6-well plates. The following day, the culture medium was replaced with 8 µL/mL of protamine sulfate (Sigma-Aldrich, P4020-5G) for 2 h. After 24 h of incubation with the virus, cells with stable knockdown were selected using puromycin (4 µl/ml for T24 cells, 3 µl/ml for TSGH 8301 cells; Cayman Chemical, Michigan, USA, 13884), as the control group was expected to be completely dead by the next day. The selected cells were then transferred to 10-cm dishes for further experiments.

### Wnt signaling pathway inhibitors

Wnt5A-derived BOX-5 peptide (BOX-5) is an N-terminally butyloxycarbonyl- (Boc) protected hexapeptide that directly binds to the FZD5. Current evidence indicates that BOX-5 exhibits high selectivity toward FZD5, though its potential off-target effects have not yet been fully characterized [36]. BOX-5 was applied to T24 and TSGH-8301 cells at a concentration of 0.75 µg/mL.

Inhibitor of Wnt production-2 (IWP-2) is a potent inhibitor of the Wnt production pathway. It functions by targeting the enzyme Porcupine (PORCN) with an IC₅₀ of 27 nM in a cell-free assay. PORCN is required for the proper palmitoylation and secretion of Wnt proteins, including Wnt3A [37]. Studies have shown that IWP-2 also inhibits Casein Kinase 1 delta (CK1δ) with an IC₅₀ of 317 nM in a cell-free assay [38, 39]. To minimize potential off-target effects resulting from CK1δ inhibition, IWP-2 was applied to T24 and TSGH-8301 cells at low concentrations of 0.625 nM and 1.25 nM, respectively.

Methyl 3-[(4-methylphenyl)sulfonyl]amino-benzoate (MSAB) is a highly potent and selective inhibitor of the Wnt/β-catenin signaling pathway. It directly binds to β-catenin, facilitating its proteasome-dependent degradation, and consequently suppresses the expression of Wnt/β-catenin target genes. MSAB exhibits minimal or no off-target effects, demonstrating low systemic toxicity and showing no impact on the growth of Wnt-independent cancer cells or normal human cells [40]. MSAB was applied to T24 and TSGH-8301 cells at concentrations of 0.25 µM and 0.125 µM, respectively.

### Statistical analysis

Data for the age-adjusted incidence rate and death rate of BC were expressed as medians with interquartile ranges, while continuous data from the cell experiments were presented as means ± SD at least three independent experiments. The Mann-Whitney U test and Kruskal-Wallis test were used to compare differences between two groups and more than two groups, respectively. Statistical analyses were conducted using GraphPad Prism, version 8.0.2 (GraphPad Software, MA, USA). Statistical significance was defined as *p* < 0.05, where * indicates *p* < 0.05, ** indicates *p* < 0.01, *** indicates *p* < 0.001, and **** indicates *p* < 0.0001.

## Results

### PM_2.5_ promotes migration and invasion of BC cells

**Fig. 1:**
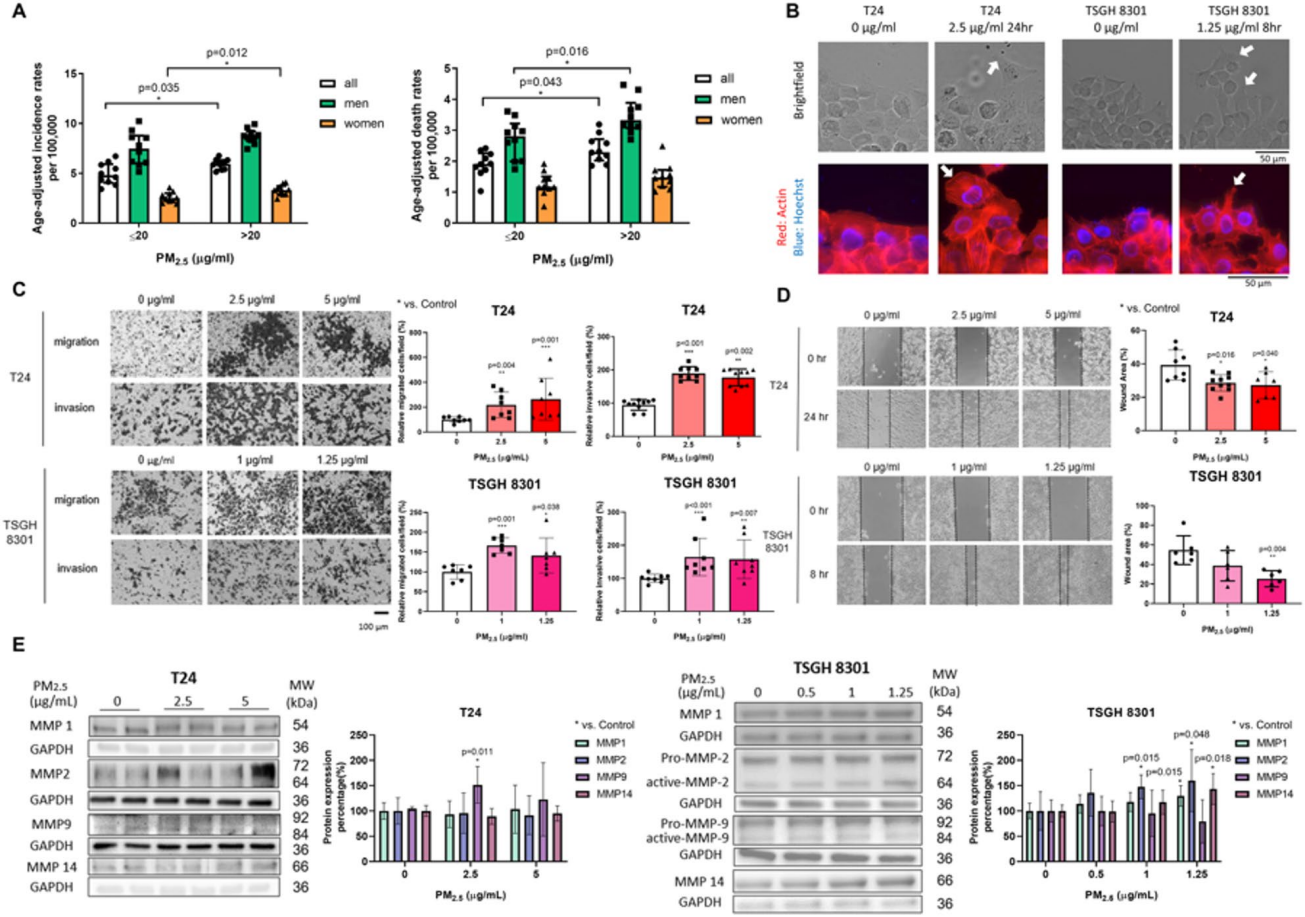
Effects of PM_2.5_ on BC migration and invasion. **A** BC age-standardized incidence and mortality rates among counties grouped by PM_2.5_ levels. **B** ICC with actin (red) and DAPI (blue) was utilized to examine the influences of PM_2.5_ on cell cytoskeleton (*white arrows*). *Scale bar*,* 50 µm.* **C** Transwell assays were performed in T24 and TSGH 8301 cells to assess the abilities of migration and invasion. The left panels showed representative fields of migrated and invaded cells exposed to different doses of PM_2.5_, 48 hours after seeding. The right graphs displayed the percentage of migrated and invasive cells per field. *Scale bar*,* 100 µm.* **D** Wound-healing assays were conducted to evaluate the migratory behavior of T24 and TSGH 8301 cells after PM_2.5_ exposure. The left panels depicted the representative fields of wound closure at 0, 24 hours for T24 cells; and 0, 8 hours for TSGH 8301 cells, respectively. The right graphs showed the percentage of residual wound area. **E** Western blot analysis and quantification for invasiveness-related protein MMPs in T24 and TSGH 8301 cells after exposure to different concentrations of PM_2.5_ for 48 hours. Data were expressed as mean ± SD. PM_2.5_: fine particulate matter 2.5; BC: bladder cancer; ICC: Immunochemistry staining; DAPI: 4’,6-diamidino-2-phenylindole; MMP: Matrix metalloproteinase. *, *p* < 0.05; **, *p* < 0.01; ***, *p* < 0.001; * represented the comparison with control group

The Ministry of Environment has established a network of air quality monitoring stations to evaluate regional air quality and long-term trends. General stations are positioned in densely populated or representative areas to reflect the air quality of the general living environment. Among the 20 counties and cities in Taiwan from 2013 to 2019, the 10 areas with PM_2.5_ exposure levels greater than 20 µg/m^3^ showed significantly higher overall BC ASIR and ASMR compared to the 10 areas with lower exposure levels (*p* = 0.035 and *p* = 0.043, respectively; Fig. 1A). In areas with higher PM_2.5_ exposure, women have a higher BC ASIR (*p* = 0.012), whereas men have a higher BC ASMR (*p* = 0.016), supporting the hypothesis of PM_2.5_ exposure in promoting BC progression. ICC, followed by wound healing assay and transwell migration/invasion assays, were performed to confirm whether PM_2.5_ affects cellular migration and invasion. T24 cells exposed to PM_2.5_ exhibited a more distinct appearance of cellular polarity and leading edge (arrows) compared to the control group (0 µg/ml) (Fig. 1B). PM_2.5_ exposure significantly increased the migration and invasion abilities in both T24 and TSGH 8301 cells, compared to the control group (Fig. 1C, D). Matrix metalloproteinases (MMPs) are extensively studied proteinases in BC that regulate tumor cell migration and invasion by degrading extracellular matrix (ECM) [41]. Expression levels of MMP1, MMP2, and MMP14 proteins were increased in TSGH 8301 cells after PM_2.5_ exposure at 1.25 µg/ml (*p* = 0.015 for MMP1, *p* = 0.048 for MMP2 and *p* = 0.018 for MMP14) (Fig. 1E). In T24 cells, only MMP9 showed a significant increase upon PM_2.5_ exposure at 2.5 µg/ml (*p* = 0.011) (Fig. 1E). These results indicated that PM_2.5_ exposure promoted the malignant behaviors in BC cells.

### RNA sequencing analysis of BC cells with PM_2.5_ exposure

**Fig. 2:**
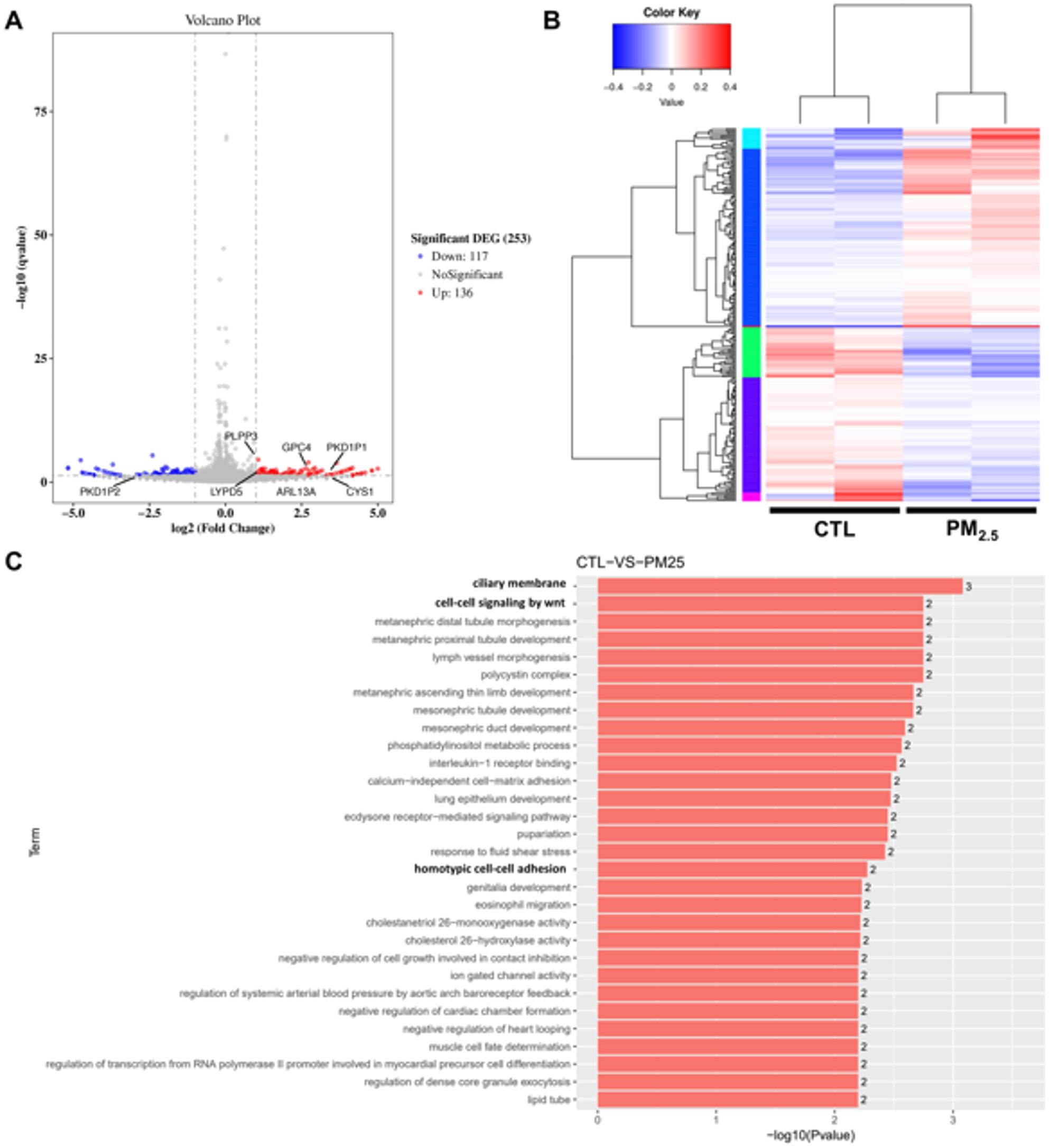
RNA sequencing for TSGH 8301 cells with or without PM_2.5_ exposure. **A** Volcano plots. Red dots symbolize genes that exhibit significant up-regulation; blue dots represent genes that display down-regulation. X axis: log2 fold change of gene expression. Y axis: statistical significance of the differential expression in log10(qvalue (fdr, padj)). **B** Differentially expressed genes were analyzed using hierarchical clustering to identify genes within the same cluster that participate in the same biological process. Clustering is performed using Log10(FPKM + 1) values. Red indicates highly expressed genes, and blue indicates low expressed genes. **C** Gene Ontology (GO) enrichment pvalue histogram. X axis: -log10(p-value) of each term. Y axis: significant enriched GO term

A total of 253 differentially expressed genes (DEGs) were identified in TSGH 8301 cells between the PM_2.5_ -exposed and unexposed group **(**Fig. 2A**)**. Significant DEGs were defined as those with a fold change greater than two (|log₂FC| > 1) and an adjusted p-value (padj) < 0.05. In comparison to the unexposed group (CTL), the PM_2.5_ exposed group showed 136 upregulated and 117 downregulated genes. These DEGs were subsequently subjected to hierarchical clustering to group genes with similar expression patterns, aiming to predict their involvement in shared signaling pathways (Fig. 2B). To further elucidate the physiological functions affected by the DEGs after PM_2.5_ exposure, Gene Ontology (GO) functional enrichment analysis showed that PM_2.5_ exposure significantly affects cell adhesion and the Wnt signaling pathway (Fig. 2C).

### PM_2.5_ activates Wnt/β-catenin signaling pathway in BC cells

**Fig. 3:**
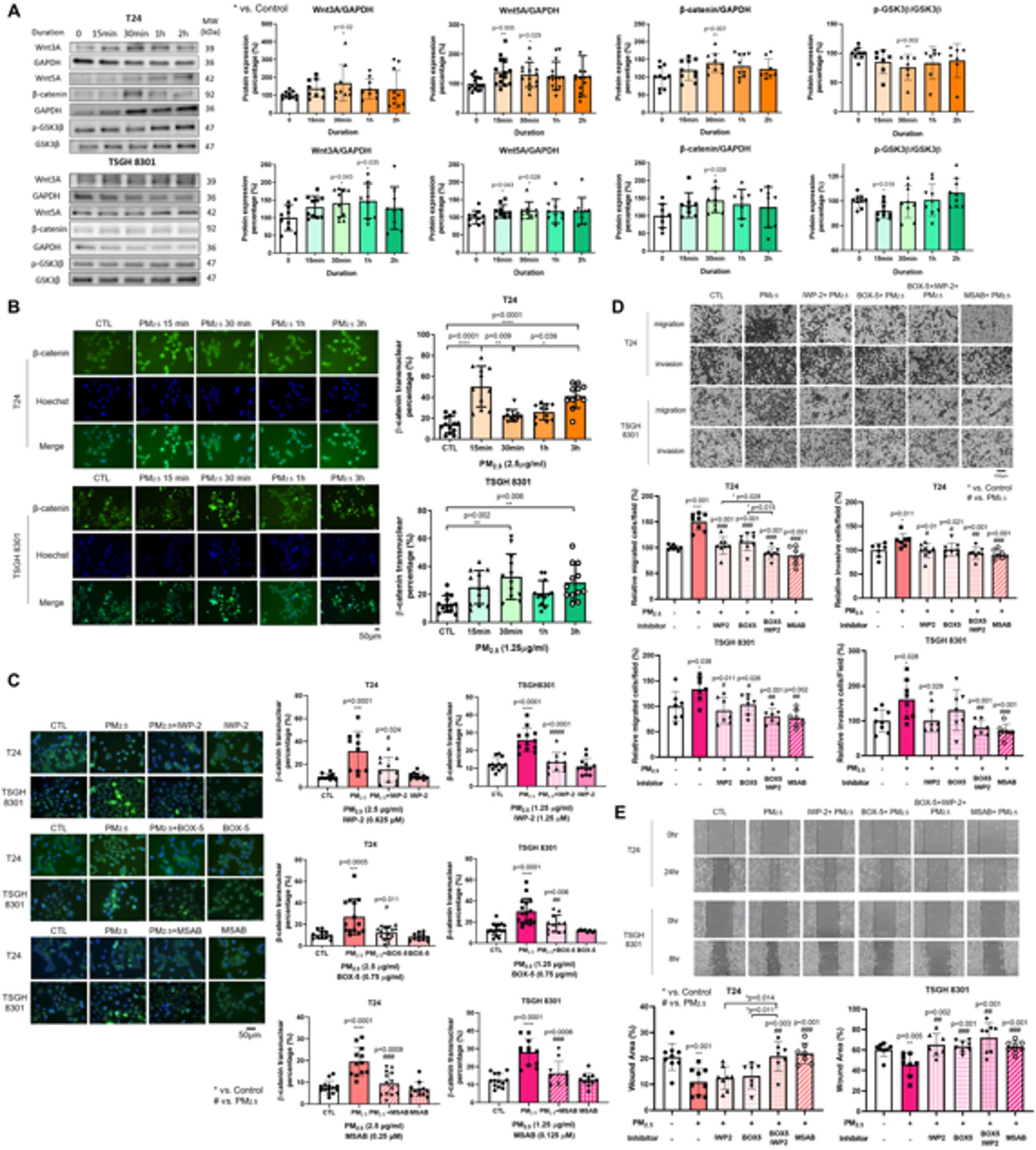
PM_2.5_ activates the Wnt/β-catenin pathway to promote migration and invasion in BC cell lines. **A** Western blot analysis showed increased expression of Wnt3A, Wnt5A, and β-catenin proteins in T24 and TSGH 8301 cells after PM_2.5_ exposure at 2.5 µg/ml and 1.25 µg/ml, respectively, for 15 min, 30 min, 1 h and 2 h. **B** ICC staining was performed with Hoechst (blue) and secondary antibodies Goat Anti-Mouse IgG Alexa Fluor^®^ 488 (green) for β-catenin to demonstrate the nuclear translocation of β-catenin in T24 and TSGH 8301 cells after PM_2.5_ exposure, for 15 min, 30 min, 1 h and 3 h. *Scale bar*,* 50 μm.* **C** ICC staining showed a suppression of β-catenin nuclear translocation in BC cells after treatment with inhibitors targeting Wnt3A, Wnt5A, and β-catenin (IWP-2: 0.625 µM for T24 cells and 1.25 µM for TSGH 8301 cells, 1 h; BOX-5: 0.75 µg/ml for both cell lines, 1 h; and MSAB: 0.25 µM for T24 cells and 0.125 µM for TSGH 8301 cells, 24 h), followed by exposure to PM_2.5_ for 30 min. *Scale bar*,* 50 μm.* **D** The transwell assays demonstrated the migratory and invasive capacities of BC cells following treatment with inhibitors for 1 h, followed by exposure to PM_2.5_. The upper panels depicted the representative images of stained migrated and invaded cells 48 h after seeding. *Scale bar*,* 100 μm*. The lower graphs show the quantification of migrated and invaded cells per field. **E** The wound healing assay demonstrated the migratory capacities of BC cells following treatment with inhibitors for 24 h, followed by exposure to PM_2.5_. The upper graphs show wound closure at 0 and 24 h for T24 cells, and at 0 and 8 h for TSGH 8301 cells. The lower graphs indicate the percentage of residual wound area at 24 h for T24 cells and 8 h for TSGH 8301 cells

To validate the results of RNA sequencing, follow-up qPCR and protein expression analyses were performed to validate the key genes affected by PM_2.5_-induced alterations. In response to PM_2.5_ exposure, qPCR analysis revealed a significant upregulation of Wnt5A and Wnt3A mRNA in T24 cells, whereas in TSGH 8301 cells, a significant increase was observed only in Wnt3A mRNA expression (Supplementary Fig. 1). Consistent with the qPCR results, PM_2.5_ exposure increased protein levels of Wnt3A, Wnt5A, and β-catenin, and reduced p-GSK3β in both T24 and TSGH 8301 cells, with statistically significant changes observed at the indicated time points (Wnt3A: *p* = 0.02 in T24 at 30 min, *p* = 0.035 in TSGH at 1 h; Wnt5A: *p* = 0.005 in T24 at 15 min and *p* = 0.028 in TSGH at 30 min; β-catenin: *p* = 0.007 in T24 and *p* = 0.028 in TSGH at 30 min; p-GSK3β: *p* = 0.002 in T24 at 30 min and *p* = 0.019 in TSGH at 15 min) (Fig. 3A). To determine whether PM_2.5_ exposure activates the Wnt/β-catenin signaling pathway, ICC staining showed that PM_2.5_ exposure significantly increased the nuclear translocation of β-catenin compared with the non-exposed CTL group (*p* < 0.0001 in T24 at 15 min, *p* = 0.002 in TSGH 8301 at 30 min) (Fig. 3B). Upon treatment with the Wnt3A, Wnt5A, and β-catenin inhibitors (IWP-2, BOX-5, and MSAB), both the nuclear translocation of β-catenin (IWP-2: *p* = 0.024 in T24, *p* < 0.0001 in TSGH; BOX-5: *p* = 0.011 in T24, *p* = 0.006 in TSGH; MSAB: *p* = 0.0009 in T24, *p* = 0.0006 in TSGH) (Fig. 3C) and the PM_2.5_-induced migratory and invasive capacities were significantly reduced compared with the PM_2.5_ exposed group. For the migration assays, significant inhibition was observed with IWP-2 (*p* < 0.001 in T24, *p* = 0.011 in TSGH), BOX-5 (*p* < 0.001 in T24, *p* = 0.026 in TSGH), and MSAB (*p* < 0.001 in T24, *p* = 0.002 in TSGH) (Fig. 3D). For the invasion assays, suppression was detected with IWP-2 (*p* = 0.01 in T24, *p* = 0.029 in TSGH), BOX-5 (*p* = 0.021 in T24), and MSAB (*p* < 0.001 in T24, *p* = 0.001 in TSGH) (Fig. 3D). Wound-healing assays similarly showed reduced motility with IWP-2 (*p* = 0.002 in TSGH), BOX-5 (*p* < 0.001 in TSGH), and MSAB (*p* < 0.001 in both T24 and TSGH), compared with PM_2.5_-exposed cells (Fig. 3E). Taken together, these results suggested that PM_2.5_ exposure promotes BC cell migration and invasion through the activation of the Wnt/β-catenin signaling pathway.

**Fig. 4:**
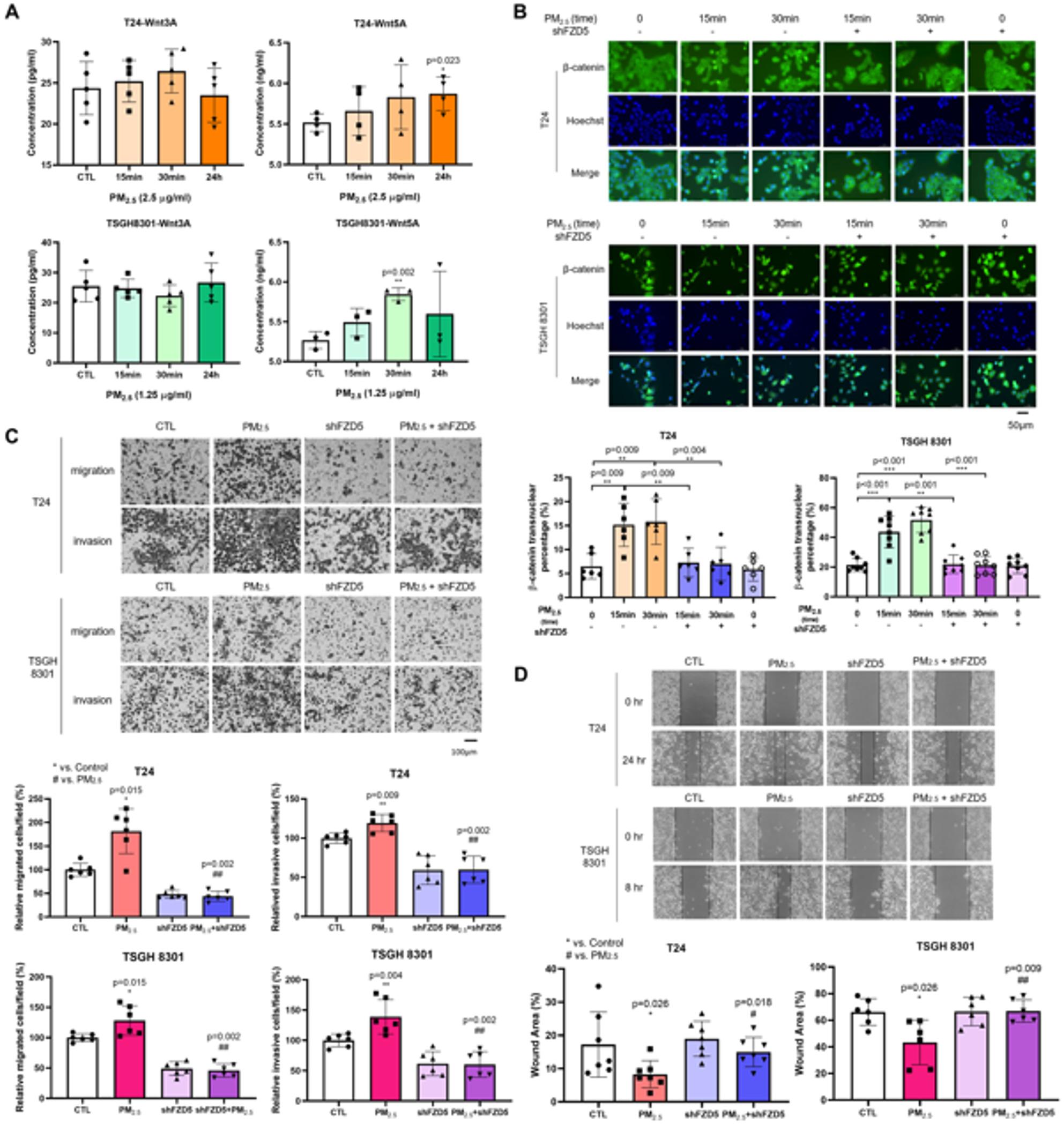
PM_2.5_ activates the Wnt/β-catenin pathway via FZD5 receptor to promote migration and invasion. **A** ELISA showed increased extracellular level of Wnt5A after PM_2.5_ exposure in T24 and TSGH 8301 cells. **B** ICC staining for β-catenin nuclear translocation was performed using Hoechst (blue) and a secondary antibody, Goat Anti-Mouse IgG Alexa Fluor^®^ 488 (green), after blocking the FZD5 receptor with shFZD in T24 and TSGH 8301 cells exposed to PM_2.5_ at 2.5 µg/ml and 1.25 µg/ml, respectively, for 15 and 30 min. *Scale bar*,* 50 μm.* **C** Transwell assays revealed the migratory and invasive capacities of T24 and TSGH 8301 cells treated with shFZD5 after exposure to PM_2.5_ at 2.5 µg/ml and 1.25 µg/ml, respectively. The upper panels depict the representative fields of stained migrated and invaded cells 48 h after seeding. *Scale bar*,* 100 μm*. The lower graphs show the quantification of migrated and invaded cells per field. **D** The wound healing assay demonstrated the migratory capacities of T24 and TSGH 8301 cells treated with shFZD5 following exposure to PM_2.5_ at 2.5 µg/ml and 1.25 µg/ml, respectively. The upper panels represent wound closure at 0 and 24 h for T24 cells, and 0 and 8 h for TSGH 8301 cells, respectively. The lower graphs indicate the percentage of residual wound area at 24 h for T24 cells and 8 h for TSGH 8301 cells, respectively. FZD5: Frizzled Family Receptor 5; ELISA: enzyme-linked immunosorbent assay. *, *p* < 0.05; **, *p* < 0.01; ***, *p* < 0.001; ****, *p* < 0.0001. * represented the comparison with control group; # represented the comparison with PM_2.5_ group

To determine whether Wnt proteins activate the β-catenin-dependent pathway extracellularly, ELISA analysis showed that extracellular Wnt5A, but not Wnt3A, was increased after PM_2.5_ exposure in both BC cell lines (T24: *p* = 0.023 at 24 h; TSGH: *p* = 0.002 at 30 min) (Fig. 4A). FZD5 has been demonstrated to mediate the Wnt/β-catenin signaling pathway in BC, and Wnt5A has been reported to display strong binding affinity to FZD5 [42]. To determine the regulatory role of the interaction between Wnt5A and FZD5, shFZD5 lentivirus was used. Similar to Wnt5A inhibition, FZD5 knockdown by shFZD5 reduced β-catenin nuclear translocation (T24: *p* = 0.004; TSGH: *p* < 0.001 at 30 min) (Fig. 4B). Significant inhibition was also observed with shFZD5 in both migration (T24 and TSGH: *p* = 0.002) and invasion assays (T24 and TSGH: *p* = 0.002) compared with PM_2.5_-exposed cells (Fig. 4C). Wound-healing assays similarly showed reduced motility following shFZD5 knockdown (T24: *p* = 0.018; TSGH: *p* = 0.009) (Fig. 4D).

### Both MAPK/ERK and Wnt/β-catenin pathways are involved in PM_2.5_-induced BC cell migration and invasion

**Fig. 5:**
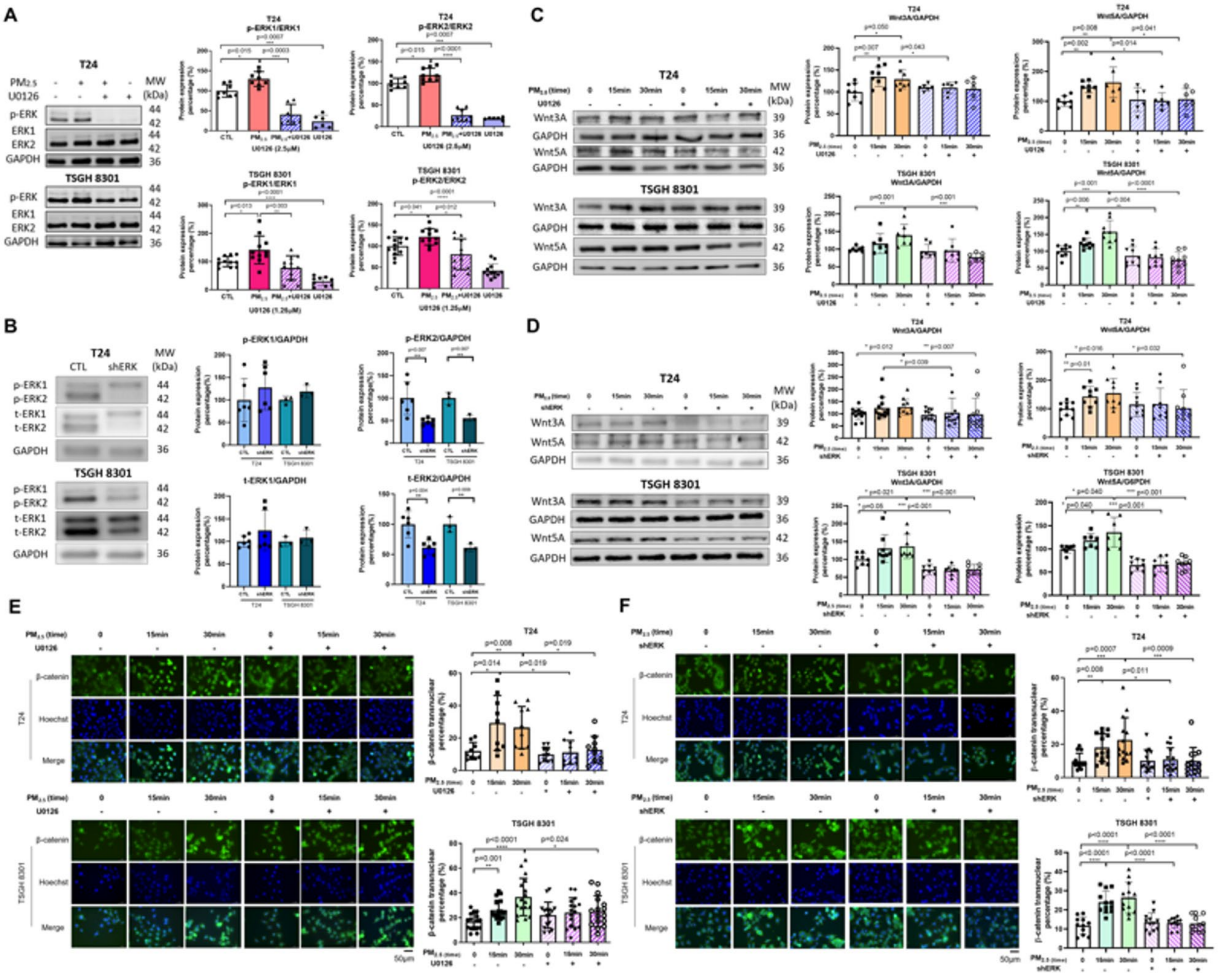
Inhibition of the MAPK/ERK pathway decreased the activation of the Wnt/β-catenin pathway after PM_2.5_ exposure. **A** Western blot analysis showed the expression levels of p-ERK1 and p-ERK2 in T24 and TSGH 8301 cells treated with the MEK inhibitor U0126 at 2.5 µM and 1.25 µM, respectively, for 1 h, or with **B** shERK, followed by exposure to PM_2.5_ at 2.5 µg/ml and 1.25 µg/ml, respectively, for 15 min. Western blot analysis showed reduced protein expression levels of Wnt3A and Wnt5A in T24 and TSGH 8301 cells treated with **C** U0126 at 2.5 µM and 1.25 µM, respectively, for 1 h, or **D** shERK, followed by exposure to PM_2.5_ at 2.5 µg/ml and 1.25 µg/ml, respectively, for 15 and 30 min. ICC showed that β-catenin nuclear accumulation was suppressed in T24 and TSGH 8301 cells treated with **E** U0126 at 2.5 µM and 1.25 µM, respectively, for 1 h, or **F** shERK, followed by exposure to PM_2.5_ at 2.5 µg/ml and 1.25 µg/ml, respectively, for 15 and 30 min. *Scale bar*, 50 μm. p-ERK1/2: 44/42 kda, titer 1:1000, Chemicon, catalog AB3826; ERK1/2: 44/42 kda, titer 1:1000, Santa Cruz, catalog sc-93/sc-154; GAPDH: 36 kda, titer 1:1000, GeneTex, catalog GTX100118; Wnt3A: 39kda, titer 1:1000, GeneTex, catalog GTX128101; Wnt5A: 42 kda, titer 1:1000, GeneTex, catalog GTX111187. Band intensities were quantified using ImageJ by measuring the integrated density of each band. The intensity of each target protein band was normalized to the corresponding loading control GAPDH to account for variations in protein loading. Relative protein expression levels were then calculated by comparing normalized values between experimental and control groups. MAPK: mitogen-activated protein kinase; ERK: extracellular regulated protein kinases. *, *p* < 0.05; **, *p* < 0.01; ***, *p* < 0.001

Cooperation between the Wnt/β-catenin and MAPK pathways is crucial in the tumorigenesis of various cancer types, including BC [21, 33]. To verify the synergistic activation of the MAPK pathway by PM_2.5_ exposure, a selective MEK inhibitor (U0126) and shERK were used to inhibit MAPK/ERK signaling. The level of phosphorylated ERK (p-ERK) represents the active status of ERK [34]. Both the inhibitor and shERK significantly decreased the PM_2.5_-induced p-ERK/ERK levels. For U0126, p-ERK1/ERK1 levels were reduced (T24: *n* = 7, *p* = 0.0003; TSGH: *n* = 8, *p* = 0.003), as were p-ERK2/ERK2 levels (T24: *n* = 9, *p* < 0.0001; TSGH: *n* = 12, *p* = 0.012) (Fig. 5A). For shERK, p-ERK2/GAPDH levels were similarly decreased (T24: *n* = 6, *p* = 0.007; TSGH: *n* = 3, *p* = 0.007) (Fig. 5B).

**Fig. 6:**
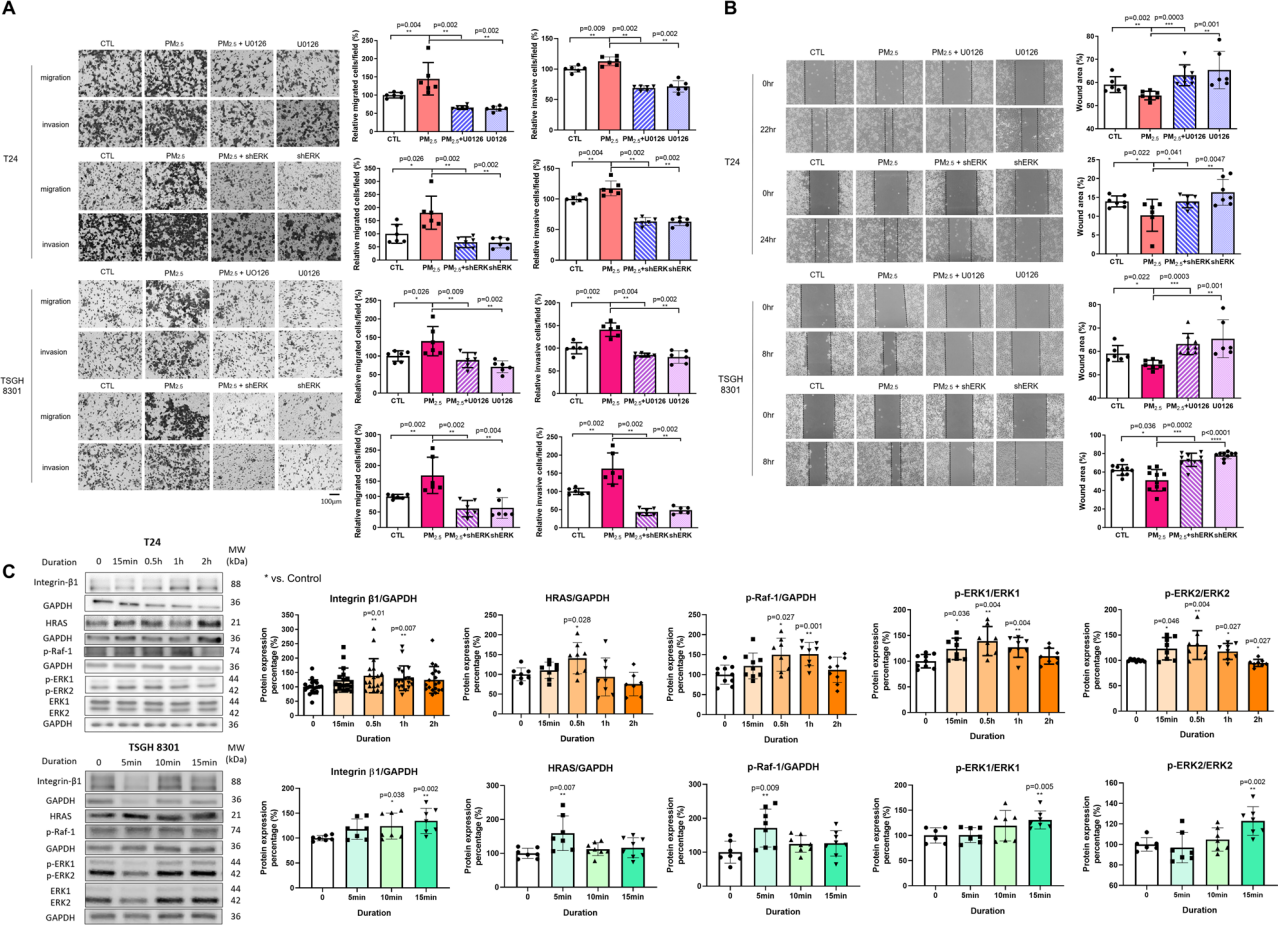
PM_2.5_ activates the MAPK/ERK pathway to enhance migration and invasion in BC cell lines. **A** Transwell assays showed the migratory and invasive capacities of T24 and TSGH 8301 cells treated with U0126 at 2.5 µM and 1.25 µM, respectively, for 1 h, or with shERK, followed by exposure to PM_2.5_ at 2.5 µg/ml and 1.25 µg/ml, respectively. The left panels depict the representative fields of stained migrated and invaded cells 48 h after seeding. *Scale bar*,* 100 μm.* The right graphs show the quantification of migrated and invaded cells per field. **B** The wound healing assay demonstrated the migratory capacities of T24 and TSGH 8301 cells after treatment with U0126 at 2.5 µM and 1.25 µM, respectively, for 1 h, or with shERK, followed by exposure to PM_2.5_ at 2.5 µg/ml and 1.25 µg/ml, respectively. The left panels show wound closure at 0 and 22/24 hours for T24 cells and at 0 and 8 h for TSGH 8301 cells, respectively. The right graphs indicate the percentage of residual wound area. **C** The Western blot assay showed increased expression of MAPK pathway proteins in T24 and TSGH 8301 cells after PM_2.5_ exposure. MAPK: mitogen-activated protein kinase; ERK: extracellular regulated protein kinases; U0126: MEK inhibitor. *, *p* < 0.05; **, *p* < 0.01; ***, *p* < 0.001; ****, *p* < 0.0001

With MEK or ERK inhibition, the PM_2.5_-induced expression of Wnt3A and Wnt5A was suppressed. For U0126 treatment, Wnt3A expression was reduced (T24: *n* = 6, *p* = 0.043 at 15 min; TSGH: *n* = 7, *p* < 0.001 at 30 min), as was Wnt5A expression (T24: *n* = 6, *p* = 0.014 at 15 min; TSGH: *n* = 9, *p* < 0.0001 at 30 min). For shERK, suppression of both Wnt3A and Wnt5A was likewise observed (T24: *n* = 12, *p* = 0.007 and *n* = 9, *p* = 0.032 at 30 min, respectively; TSGH: *n* = 8, both *p* < 0.001 at 15 min) **(**Fig. 5C, D). Co-immunoprecipitation (Co-IP) analysis revealed that, following PM_2.5_ exposure, ERK co-precipitated with GSK3β, Wnt3A, and Wnt5A, indicating its association with upstream Wnt components (Supplementary Fig. 2). Through MEK or ERK inhibition, PM_2.5_-induced β-catenin nuclear translocation was suppressed. For U0126, this reduction was evident in T24 cells (*n* = 9, *p* = 0.019 at 15 min) and TSGH cells (*n* = 16, *p* = 0.024 at 30 min). For shERK, nuclear β-catenin levels were similarly decreased in T24 cells (*n* = 13, *p* = 0.0009 at 30 min) and in TSGH cells (*n* = 11, *p* < 0.0001 at 15 min) (Fig. 5E and F). Consistently, both U0126 and shERK markedly reduced the PM_2.5_-induced migratory and invasive abilities of T24 and TSGH 8301 cells. In transwell migration assays, U0126 significantly decreased cell migration (T24: *n* = 6, *p* = 0.002; TSGH: *n* = 6, *p* = 0.009), with shERK showing comparable effects (both cell lines *n* = 6, *p* = 0.002) (Fig. 6A). Wound-healing assays likewise demonstrated reduced PM_2.5_-driven motility following U0126 treatment (both cell lines *n* = 8, *p* = 0.0003), and shERK produced similar inhibition (T24: *n* = 6, *p* = 0.041; TSGH: *n* = 10, *p* = 0.0002) (Fig. 6B). In invasion assays, U0126 suppressed invasive capacity (T24: *n* = 6, *p* = 0.002; TSGH: *n* = 6, *p* = 0.004), and shERK again yielded comparable reductions (both cell lines *n* = 6, *p* = 0.002) (Fig. 6A). The raw cell counts corresponding to the migration and invasion assays display the same trends as the quantified data and are provided in Supplementary Fig. 3. Since bidirectional crosstalk between Wnt/β-catenin and MAPK/ERK signaling has been described in previous studies, we further found that Wnt inhibition prevented PM_2.5_-induced ERK activation, indicating bidirectional crosstalk between these pathways (Supplementary Fig. 4) [33]. While PM_2.5_-activated ERK enhances Wnt ligand expression and β-catenin signaling, ERK activation also depends on upstream Wnt signaling. The loss of ERK phosphorylation upon Wnt blockade demonstrates that the two pathways mutually reinforce each other.

Since integrins are crucial receptors that mediate cell-cell and cell-ECM interactions, facilitating cell migration and invasion by activating MAPK/ERK pathway in BC, the integrin-related signaling pathway was analyzed [43]. The protein levels of integrin β1, HRAS, p-Raf-1, p-ERK1 and p-ERK2 were all significantly increased in both BC cell lines after PM_2.5_ exposure (Fig. 6C). These results suggested that PM_2.5_ activates MAPK/ERK pathway and the downstream Wnt/β-catenin pathway, thereby enhancing the malignant behavior of BC cells.

**Fig. 7:**
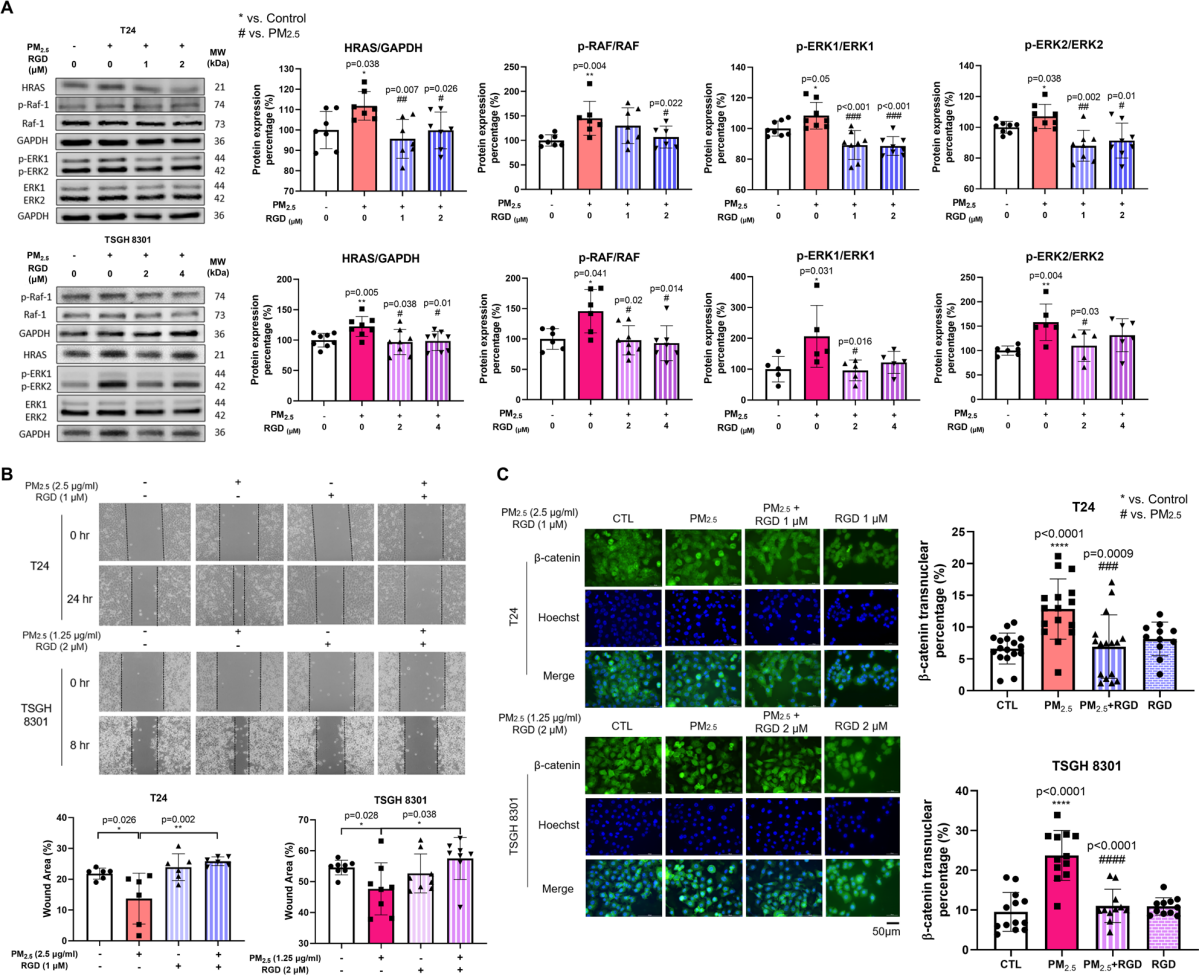
PM2.5 exposure promotes BC progression by activating the MAPK/ERK pathway via integrinβ1. (**A**) Western blot analysis showed reduced protein expression levels of HRAS, p-RAF, p-ERK1, and p-ERK2 in T24 and TSGH 8301 cells treated with RGD peptide for 24 hours, followed by exposure to PM2.5 at 2.5 μg/ml and 1.25 μg/ml, respectively, for 30 minutes. (**B**) The wound healing assay revealed a reduction in the migration capacity of T24 and TSGH 8301 cells after treatment with RGD at 1 μM and 2 μM, respectively, for 24 hours, followed by exposure to PM2.5. The upper panels show wound closure at 0 and 24 hours for T24 cells and at 0 and 8 hours for TSGH 8301 cells. The lower graphs indicate the percentage of residual wound area. (**C**) ICC demonstrated that β-catenin nuclear accumulation was suppressed in T24 and TSGH 8301 cells treated with RGD peptide at 1 μM and 2 μM, respectively, for 24 hours, followed by exposure to PM2.5. Scale bar, 50 μm. MAPK: mitogen-activated protein kinase; ERK: extracellular regulated protein kinases; RAS: Rat Sarcoma; RAF: Rapidly Accelerated Fibrosarcoma; RGD: Arg-Gly-Asp peptide motif; ICC: Immunochemistry staining. *, p< 0.05; **, p< 0.01; ***, p< 0.001; ****, p< 0.0001. * represented the comparison with control group; # represented the comparison with PM2.5 group

As a potent integrin ligand, the Arg-Gly-Asp (RGD) peptide motif mediates the binding of cells to the ECM, thereby regulating crucial biological functions in cancer cells, such as cellular adhesion, migration, and signaling pathways [44]. A synthetic RGD ligand targeting integrin β1 was used to inhibit integrin signal transduction. The expression levels of HRAS, p-Raf-1, p-ERK1 and p-ERK2, which were stimulated by PM_2.5_ exposure, decreased upon RGD treatment (Fig. 7A). In addition, the wound healing assay demonstrated that blockade of integrin β1 significantly reduced the migratory capacity of PM_2.5_-exposed BC cells (*p* = 0.002 in T24 and *p* = 0.038 in TSGH) (Fig. 7B). The PM_2.5_-induced nuclear translocation of β-catenin was also diminished in BC cells treated with RGD (*p* = 0.0009 in T24 and *p* < 0.0001 in TSGH) (Fig. 7C). Overall, our results supported that PM_2.5_ promotes BC cell migration and invasion through integrin-mediated activation of the MAPK/ERK pathway, followed by the Wnt/β-catenin pathway.

## Discussion

**Fig. 8:**
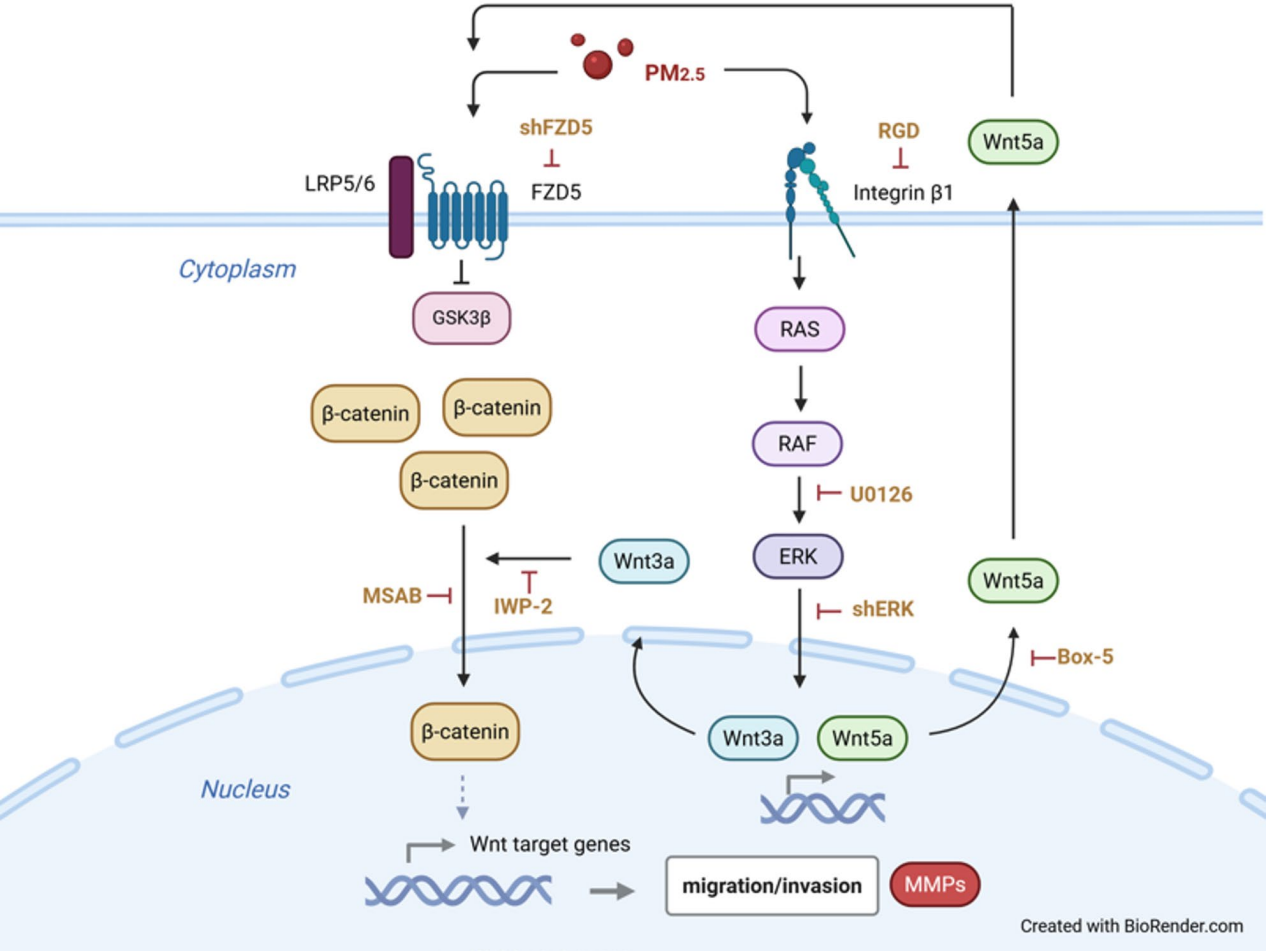
Schematic summary. PM_2.5_ exposure enhanced BC cell migration and invasion through the integrin-mediated MAPK/ERK pathway, which subsequently activated the Wnt/β-catenin signaling pathway. Inhibition of MEK or ERK reduced Wnt protein expression and β-catenin nuclear translocation, confirming the upstream role of MAPK/ERK. PM_2.5_: fine particulate matter 2.5; BC: bladder cancer; MAPK: mitogen-activated protein kinase; ERK: extracellular regulated protein kinases; LRP: low-density lipoprotein receptor-related protein; Fzd: Frizzled; IWP-2: inhibitor of Wnt production-2; MSAB: methyl 3-[(4-methylphenyl)sulfonyl]amino-benzoate; RAS, Rat Sarcoma; RAF, Rapidly Accelerated Fibrosarcoma; RGD: Arg-Gly-Asp peptide; BOX-5: Wnt5A-derived Box-5 peptide; MMPs: Matrix metalloproteinases. Created in BioRender. NTU, A. (2025) https://BioRender.com/lmntiwe

BC is strongly linked to a variety of environmental factors, many of which involve exposure to chemicals or toxins [8, 32, 45]. Among air pollution components, PM_2.5_ exposure has been shown to enhance lung carcinogenic properties in both lung normal bronchial epithelial cell and lung cancer cell lines, leading to increased cell proliferation, migration and invasion [46]. Although studies have reported a positive association between air pollution and BC, inconsistent results in cancer incidence and mortality highlight certain limitations, including poor adjustment for confounders and insufficient follow-up time [3, 9, 15, 47]. Currently, no direct evidence demonstrates the exact pathophysiology of PM_2.5_ exposure on BC cells. In this study, we revealed that PM_2.5_ exposure aggravated BC cell migration and invasion by activating both the MAPK/ERK and Wnt/β-catenin signaling pathways (Fig. 8). Inhibition of the MAPK/ERK pathway prevented the activation of the PM_2.5_-triggered Wnt/β-catenin pathway, indicating that the MAPK/ERK pathway acts upstream of Wnt/β-catenin signaling. We confirmed that PM_2.5_ stimulated an increase in Wnt3A and Wnt5A, with Wnt5A acting extracellularly by binding to the FZD receptor, leading to the nuclear translocation of β-catenin. Although Wnt5A is primarily classified as a non-canonical ligand, our data showed no activation of the downstream effectors of either the planar cell polarity (PCP) or Wnt/Ca^2+^ pathways, including cell division control protein 42 homolog (Cdc42), Ras homolog family member A (RhoA), Ras-related C3 botulinum toxin substrate (Rac) and JNK (Supplementary Fig. 5).

Cell migration and invasion are hallmarks of malignant transformation in cancer cells and are crucial for metastasis formation. These processes involve profound morphological changes, including the formation of protrusions, dynamic cytoskeletal rearrangements, polarization, focal adhesion formation, and ECM degradation, all of which enable cancer cells to move through tissues [48]. MMPs, as zinc-dependent endopeptidases, play a key role in cancer invasion and metastasis by breaking down ECM barriers and facilitating cell movement [41]. PM_2.5_ has been reported to induce migration and invasion by upregulating MMPs in lung cancer cells [49, 50]. MMPs have also been extensively studied in BC, where increased MMPs production correlates with a more invasive phenotype and poorer clinical outcomes [51]. In this study, PM_2.5_ exposure increased the expression levels of MMP-1, MMP-2, and MMP-14 proteins in TSGH 8301 cells, while only MMP-9 was upregulated in T24 cells. This discrepancy between cell lines may be attributed to differences in MMP expression patterns across distinct cancer types or stages, influenced by complex regulatory mechanisms [52]. Moreover, there are still conflicting reports regarding whether MMPs are primarily produced by tumor cells or stromal cells, which contribute to the dynamic microenvironment [51]. Roomi et al. reported that MMP-9 expression in T24 cells was stimulated by the cytokine pathway despite its low basal level [53]. Therefore, the expression of MMPs in tumor specimens may hold greater clinical significance than results obtained from a limited number of cell lines.

The Wnt signaling pathway plays a critical role in promoting self-renewal, cell proliferation, and differentiation of cancer stem cells, as well as driving the EMT process, all of which contribute to tumorigenesis and cancer metastasis [54]. Li et al. showed that PM_2.5_ activates the Wnt/β-catenin pathway in lung carcinogenesis, and chronic PM_2.5_ exposure similarly induced Wnt/β-catenin activation in rat lungs, supporting its potential carcinogenic effects [46, 55]. Additionally, Wnt3A-enriched exosomes secreted by lung cancer cells after PM_2.5_ exposure activated the Wnt/β-catenin signaling pathway, promoting cell proliferation and xenograft tumor growth [28]. Furthermore, the Wnt5A gene was upregulated in a bronchial epithelial cell line in response to PM_2.5_ exposure and contributes to cancer progression and metastasis by regulating cell migration, polarity, and cytoskeletal dynamics [56]. Although Wnt5A is primarily recognized as a non-canonical ligand, it has been shown to activate canonical signaling depending on the cellular context and receptor availability [56]. Wnt5A plays a dual role in cancer, acting as a tumor promoter in some cancers, such as ovarian and nasopharyngeal cancers, by inducing EMT and cell migration, while acting as a tumor suppressor in others, like gastric and CRC, by inhibiting EMT. In BC, Wnt5A has been reported to exacerbate disease progression by activating the canonical Wnt signaling pathway [24]. In this study, we observed that both Wnt3A and Wnt5A were upregulated upon PM_2.5_ exposure, promoting cell migration and invasion via the Wnt/β-catenin signaling pathway. Inhibition of either Wnt3A or Wnt5A with IWP-2 or BOX-5, respectively, effectively suppressed PM_2.5_-induced migration and invasion in TSGH 8301 cells. Although both inhibitors were able to reduce PM_2.5_-induced migration and invasion in T24 cells in transwell assays, neither BOX-5 nor IWP-2 alone was sufficient to inhibit the migration in the wound healing assay. Instead, both inhibitors needed to be used in combination to achieve this effect. As the T24 cell line originates from highly malignant and poorly differentiated BC, Malgor et al. demonstrated that Wnt5A mRNA expression was markedly reduced in T24 cells compared to normal urothelial cells [23]. The application of Wnt5A-conditioned medium significantly enhanced the migration of T24 cells compared to regular and control-conditioned media. Nevertheless, the addition of recombinant Wnt5A did not notably affect T24 cell migration. This suggested that additional signaling molecules, such as Wnt3A, are also required simultaneously to modulate migration in T24 cells.

An increasing number of studies highlight the Wnt/β-catenin pathway as a potential therapeutic target that activates downstream genes [57, 58]. Our findings support Wnt/β-catenin as a key mediator of PM_2.5_-induced cancer progression in BC, suggesting that inhibitors targeting this pathway may offer therapeutic potential. Agents such as the FZD5-targeting antibodies OMP-18R5 (vantictumab) and IgG-2919, as well as β-catenin inhibitors including CWP232291, CGP049090, LF3, MSAB, PKF115-584, PKF118-310, ICG-001, and SAH-BLC9, represent candidates for further development [40, 59–66]. Immunotherapy also plays a central role in advanced BC, with PD-1/PD-L1 inhibitors demonstrating significant clinical benefit [67–69]. The Wnt/β-catenin signaling pathway critically shapes the tumor immune microenvironment by activating dendritic-cell β-catenin, promoting Treg activity, and suppressing CD8⁺ T-cell responses [70, 71]. Aberrant pathway activation thereby supports immune evasion. Thus, targeting Wnt signaling may not only offer a therapeutic strategy for PM_2.5_-associated BC but also enhance the efficacy of existing immune checkpoint inhibitors [72].

Previous studies indicate that BC is highly dependent on the MAPK/ERK pathway [73]. The interaction between integrins and the MAPK/ERK pathway plays a crucial role in cancer migration and invasion by regulating cytoskeletal dynamics, ECM degradation, and EMT [74]. The MAPK/JNK signaling pathway was not a major activation pathway following PM_2.5_ exposure in either T24 or TSGH8301 cells, whereas activation of the MAPK/p38 pathway was only observed in TSGH 8301 cells (Supplementary Fig. 5). The MAPK/p38 pathway has been reported to play a lesser role in the cell cycle of advanced BC cells [75]. The differences between these cell lines underscore the presence of distinct signaling mechanisms among cancer cells of different grades.

Integrins are heterodimeric glycoproteins on the cell surface, composed of at least 18 α subunits and 8 β subunits, forming more than two dozen distinct integrins [76]. Upon binding to ECM proteins, integrins activate the RAS-RAF-MEK-ERK cascade, promoting cytoskeletal reorganization and facilitating focal adhesion turnover and actin polymerization, both of which are essential for cell motility [74]. In the present study, we found that PM_2.5_-induced cell migration and invasion were associated with the activation of the MAPK/ERK pathway. Inhibition of this pathway using U0126 or shRNA reversed PM_2.5_-triggered cell migration and invasion. Furthermore, treatment with a synthetic RGD ligand targeting integrin β1 in PM_2.5_-exposed BC cells significantly reduced the expression of key signaling proteins in the MAPK/ERK pathway, thereby decreased both migration and β-catenin nuclear translocation. These findings suggested a crucial role for integrin-mediated ERK activation in PM_2.5_-induced BC progression. Approximately 45% of therapeutic targets identified in BC involve the MAPK pathway, underscoring its importance as a therapeutic focus [77]. Several MEK inhibitors—including cobimetinib, trametinib, binimetinib, and selumetinib—are FDA-approved, while ERK inhibitors such as ulixertinib (BVD-523), GDC-0994, LY3214996, MK-8353, and SCH772984 have been developed to directly block the terminal kinase and potentially overcome resistance to upstream inhibitor [78–86]. Tyrosine kinase inhibitors (TKIs) that act on the MAPK/ERK pathway, such as regorafenib, have also demonstrated clinical activity; regorafenib not only induces apoptosis but suppresses MAPK/NF-κB–mediated tumor progression in BC [87, 88]. Growing evidence further indicates that MAPK activation promotes immune evasion and resistance to PD-1/PD-L1 inhibitors, suggesting that targeting this pathway may offer a promising therapeutic strategy for PM_2.5_-related BC [89].

The interplay between the MAPK/ERK pathway and Wnt/β-catenin signaling is crucial for both normal cellular functions and cancer progression, yet the outcomes of their interaction vary greatly across different cancer types. In CRC, Wnt/β-catenin signaling inhibits GSK3β-mediated phosphorylation and degradation of H-Ras, leading to Ras stabilization and contributing to intestinal tumorigenesis [35]. Moreover, expression of MAPK target genes is enriched in highly Wnt-active CRC cells. In melanoma, MAPK/ERK and Wnt/β-catenin signaling exhibit negative crosstalk. BRAFV600 inhibition enhances β-catenin activity to promote cell apoptosis, accompanied by AXIN1 degradation and GSK3β inhibition. Conversely, hyperactivated MAPK/ERK signaling stabilizes AXIN1 and suppresses Wnt signaling [90]. Recent evidence suggests that the interaction between these pathways is bidirectional. MAPK activation can also potentiate Wnt signaling, as shown by the colocalization of nuclear β-catenin with phospho-ERK, and the correlation between nuclear β-catenin and KRAS mutations [91]. With concurrent APC and KRAS mutations, MAPK signaling promotes nuclear β-catenin accumulation and Wnt target gene expression, forming a positive feedback loop in CRC [33]. In BC, a significant correlation was observed between β-catenin upregulation and pERK1/2 activation [21]. Neither Ras mutation nor β-catenin activation alone induces BC, but the combination accelerates tumor formation and confers sensitivity to MEK inhibition. To further examine the bidirectional crosstalk between the two signaling pathways, Western blot analysis demonstrated that ERK inhibition suppressed PM_2.5_-induced Wnt ligand expression. Co-IP analysis also showed that ERK pulled down GSK3β, WNT3A, and WNT5A after PM_2.5_ treatment, indicating its association with upstream Wnt components (Supplementary Fig. 2). Although β-catenin was not detected, this may be due to its nuclear translocation. We also used Wnt inhibitors and found that, in the absence of Wnt signaling, PM_2.5_ failed to activate ERK (Supplementary Fig. 4), further supporting the bidirectional interaction between the two pathways.

Currently available evidence investigating the relationship between PM_2.5_ exposure and BC is mostly based on studies with various exposure metrics, analytical methods, and research designs [3]. Accurately assessing individual exposure to PM_2.5_ over extended periods is challenging. Many studies rely on residential addresses to estimate exposure, which may not account for variations in pollution levels across different locations and times. Meanwhile, BC shares risk factors with other diseases, notably smoking [6]. Disentangling the effects of PM_2.5_ exposure from smoking and other variables is complex. Since cancer development is a prolonged and gradual process, establishing a causal relationship between PM_2.5_ and BC remains difficult. To our knowledge, this study provides the first molecular evidence of PM_2.5_-induced cancer progression in BC. Cell models allow precise regulation of experimental conditions, such as PM_2.5_ concentration and exposure duration, ensuring reproducibility and mechanistic insights. However, several limitations exist in this study. Traditional 2D cell cultures fail to replicate the tumor microenvironment and immune system interactions. Additionally, in vitro studies with short-term or high-dose exposures may not accurately reflect real-world effects. Lastly, PM_2.5_ sources and components may vary by location, season, and pollution source, making standardization across studies more challenging.

## Conclusions

In summary, PM_2.5_ exposure promoted the migratory and invasive abilities in BC cells via activation of the integrin-mediated MAPK/ERK pathway, which, in turn, stimulated the Wnt/β-catenin pathway. Inhibition of the MAPK/ERK pathway downregulated the expression of Wnt proteins and subsequent β-catenin nuclear translocation, demonstrating that the MAPK/ERK pathway functions upstream in activating the Wnt/β-catenin pathway. Overall, our findings not only contribute to a better understanding of PM_2.5_-related-BC progression but also offer a potential therapeutic target for environmental factors.

## Supplementary Information

Supplementary Material 1

## Data Availability

Data will be made available on reasonable request. Publicly available statistics on PM 2.5 levels were obtained from the Environmental Protection Administration, Executive Yuan, while age-adjusted incidence and mortality rates were retrieved from the Cancer Registry Database maintained by the Ministry of Health and Welfare and the National Health Insurance Administration.

## Abbreviations

Akt: Ak strain transforming
APC: Adenomatous polyposis coli
AXIN: Axis inhibition protein
ASIR: Age-standardized incidence rates
ASMR: Age-standardized incidence mortality rates
APS: Ammonium persulfate
BC: Bladder cancer
BSA: Bovine serum albumin
BOX-5: Wnt5A-derived Box-5 peptide
CK1: Casein kinase 1
cDNA: Complementary deoxyribonucleic acid
Cdc42: Cell division control protein 42 homolog
c-Myc: Myelocytomatosis oncogene cellular homolog
Co-IP: Co-immunoprecipitation analysis
CRC: colorectal cancer
DVL: Dishevelled
DAG: Directed Acyclic Graph
DMEM: Dulbecco’s Modified Eagle Medium
ddH₂O: Double-distilled water
DEGs: Differentially expressed genes
EDTA: Ethylenediaminetetraacetic acid
ERK: Extracellular signal-regulated kinase
ECL: Enhanced chemiluminescent reagents
ELISA: Enzyme-linked immunosorbent assay
ECM: Extracellular matrix
EMT: Epithelial-mesenchymal transition
Fzd: Frizzled
FBS: Fetal Bovine Serum
GO: Gene Ontology
IARC: International Agency for Research on Cancer
ICC: Immunocytochemistry
IWP-2: Inhibitor of Wnt production-2
GSK-3β: glycogen synthase kinase 3β
JNKs: c-Jun N-terminal kinases
KEGG: Kyoto Encyclopedia of Genes and Genomes
LRP: Low-density lipoprotein receptor-related protein
MAPK: Mitogen-activated protein kinase
MEK: MAPK kinase
M.O.I: Multiplicity of infection
MMPs: Matrix metalloproteinases
MSAB: Methyl 3-[(4-methylphenyl)sulfonyl]amino-benzoate
NIST: National Institute of Standards and Technology
OD: Optical density
Vantictumab: OMP-18R5
PM_2.5_: Fine particulate matter 2.5
PAHs: Polycyclic aromatic hydrocarbons
PI3K: Phosphatidylinositol 3-kinase
PCR: Polymerase chain reaction
PCP: Planar cell polarity
RNA: Ribonucleic acid
RAS: Rat Sarcoma
RAF: Rapidly Accelerated Fibrosarcoma
RNA-seq: RNA sequencing
RGD: Arg-Gly-Asp peptide
RhoA: Ras homolog family member A
Rac: Ras-related C3 botulinum toxin substrate
SNPs: Single-nucleotide polymorphisms
SRM: Standard Reference Material
SDS-PAGE: Sodium dodecyl sulfate–polyacrylamide gel electrophoresis
SABC: Streptavidin-biotin complex
shRNA: Short hairpin RNA
TSGH 8301: Tri-Service General Hospital 8301
TEMED: Tetramethylethylenediamine
TBST: Tris-buffered saline containing 0.1% Tween 20
TMB: 3,3’,5,5’-tetramethylbenzidine
UHVS: Ultra-high-volume sampler
U0126: Selective MEK inhibitor
Wnt: Wingless-related integration site
WHO: World Health Organization
ZEB1: Zinc finger E-box binding homeobox 1
