# Supplementary material for: Particulate matter 2.5 promotes bladder cancer cell migration and invasion through the crosstalk between integrin-mediated MAPK/ERK and Wnt/β-catenin pathways
Source: Part Fibre Toxicol. 2026 Jan 16;23:4. doi: 10.1186/s12989-025-00656-3 (PMC12828959; doi:10.1186/s12989-025-00656-3)
Supplement: Supplementary file 1 — Supplementary Material 1 [file 12989_2025_656_MOESM3_ESM.zip › Supplementary material 1/Supplementary Figures.docx]

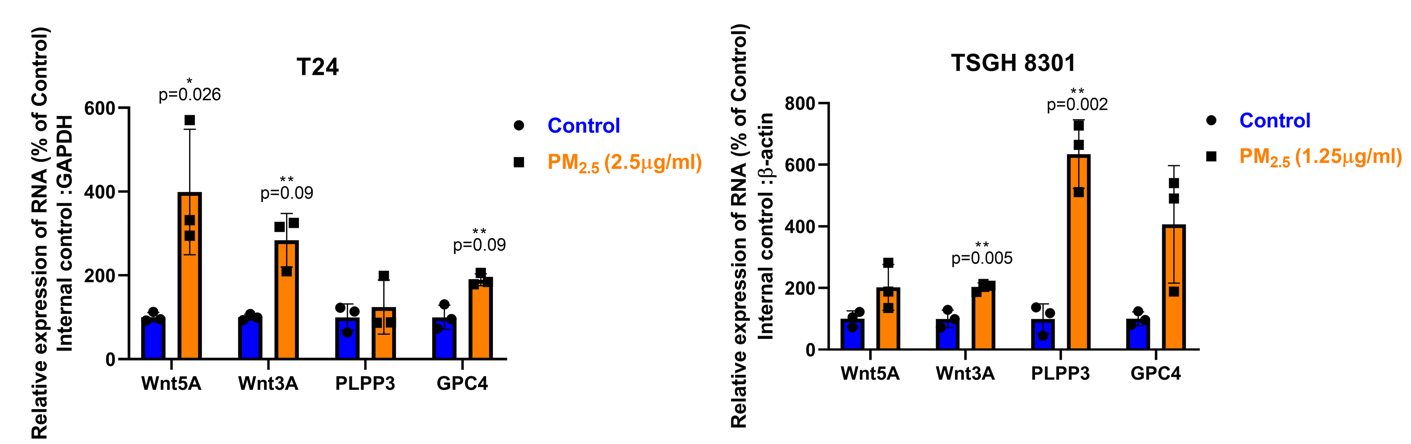


**Supplementary Figure 1**


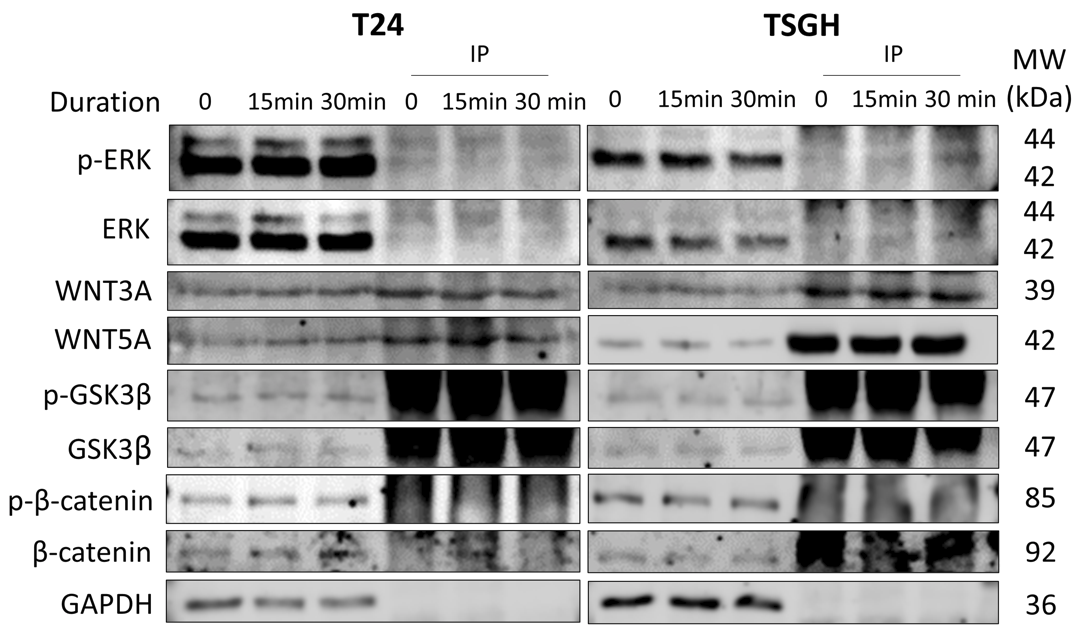


**Supplementary Figure 2**


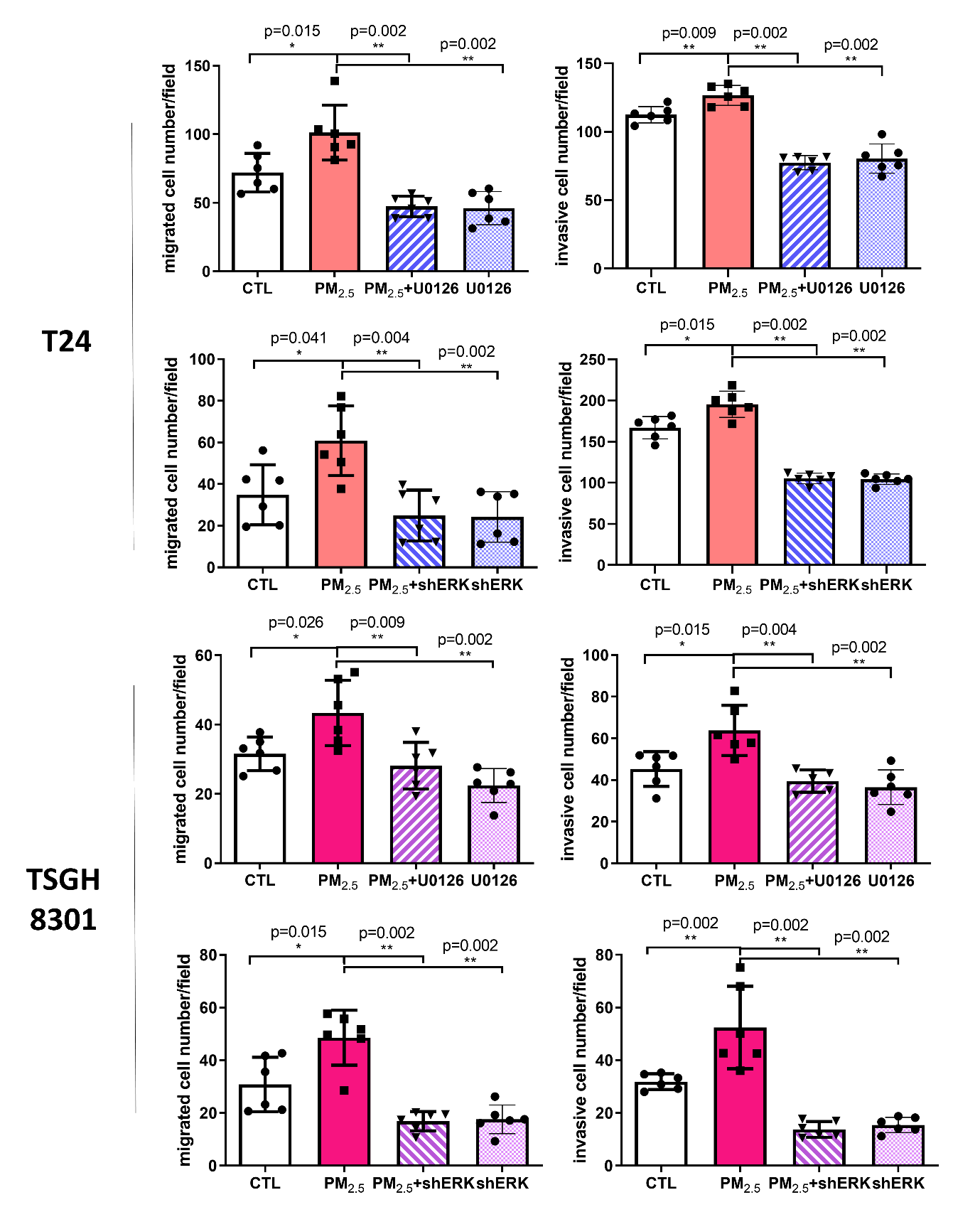


**Supplementary Figure 3**


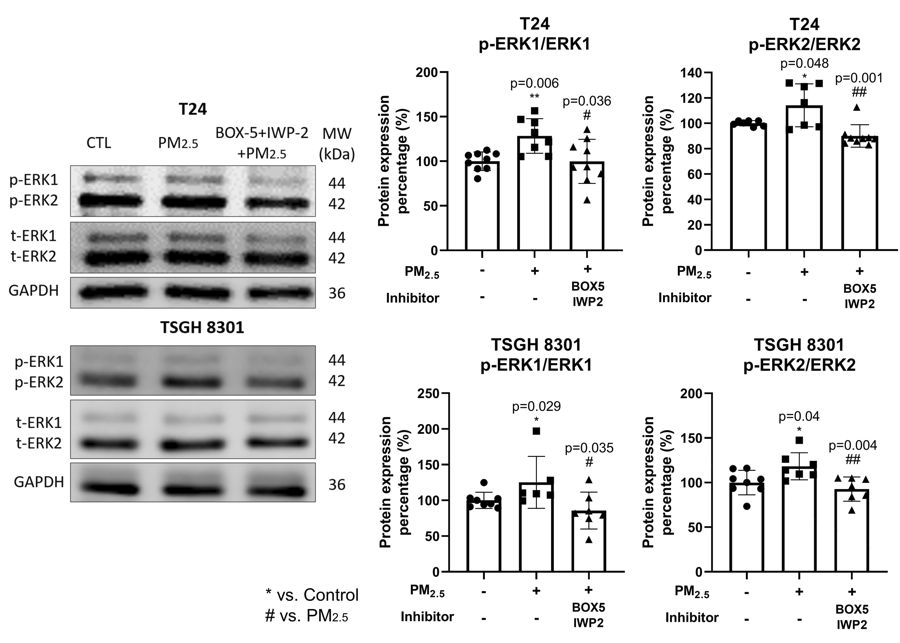


**Supplementary Figure 4**


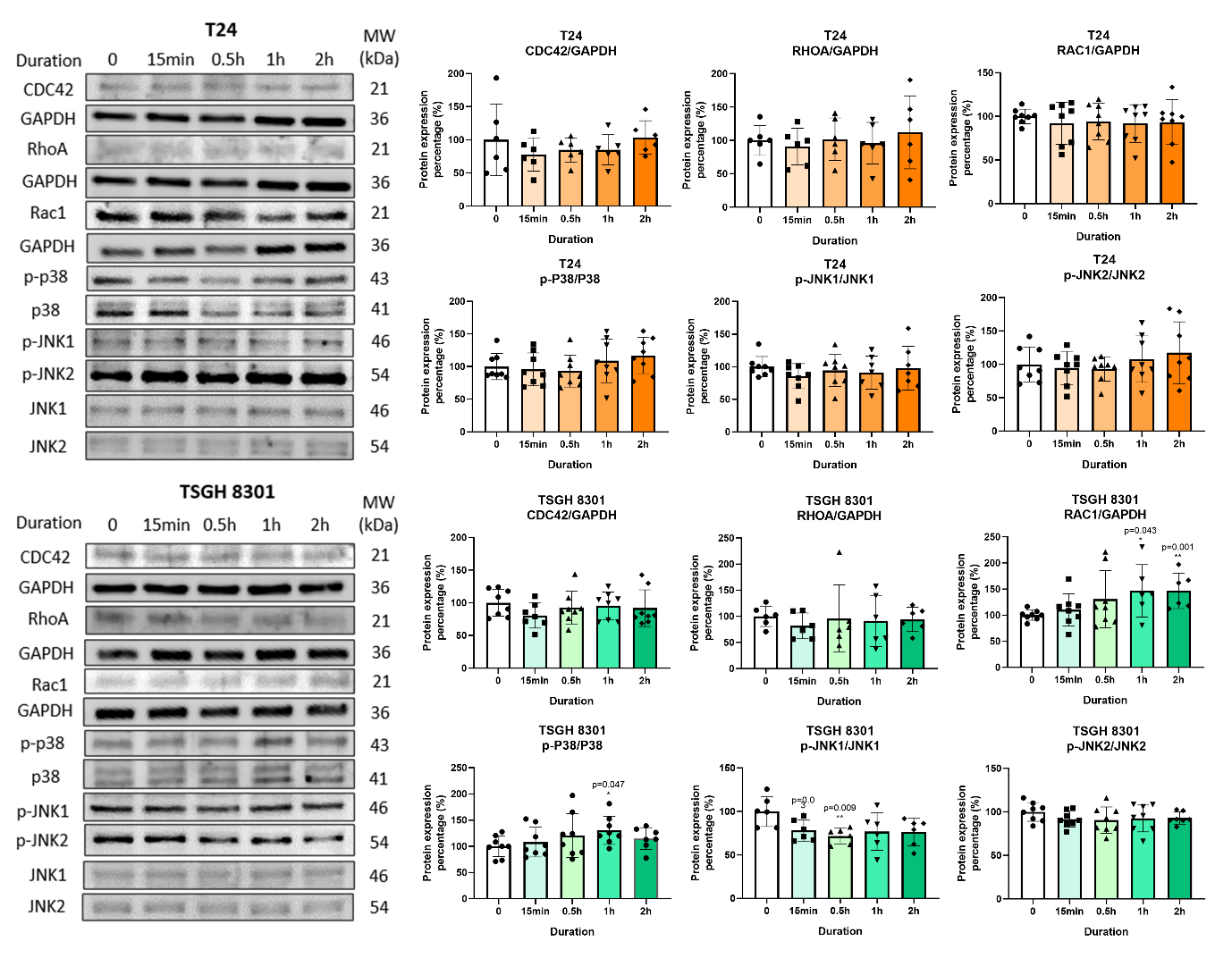


**Supplementary Figure 5**
