# Supplementary material for: Particulate matter 2.5 promotes bladder cancer cell migration and invasion through the crosstalk between integrin-mediated MAPK/ERK and Wnt/β-catenin pathways
Source: Part Fibre Toxicol. 2026 Jan 16;23:4. doi: 10.1186/s12989-025-00656-3 (PMC12828959; doi:10.1186/s12989-025-00656-3)
Supplement: Supplementary file 1 — Supplementary Material 1 [file 12989_2025_656_MOESM3_ESM.zip › Supplementary material 1/Supplementary Figure captions.docx]

**Supplementary Figure captions:**

**Supplementary Fig. 1.** Validation of differentially expressed genes identified by RNA sequencing using qPCR.

**Supplementary Fig. 2**. Co-immunoprecipitation analysis (Co-IP) of ERK-associated Wnt pathway components in T24 and TSGH 8301 cells following PM_2.5_ exposure. Co-IP assays were performed to examine the interaction between ERK and upstream Wnt pathway components after PM_2.5_ treatment for 15 and 30 minutes. ERK immunoprecipitation successfully pulled down GSK3β, Wnt3A, and Wnt5A in both T24 and TSGH 8301 cells, indicating that PM_2.5_ enhances the association of ERK with key regulators of the Wnt/β-catenin signaling pathway.

**Supplementary Fig. 3**. PM_2.5_ activates the MAPK/ERK pathway to enhance migration and invasion in BC cell lines. The raw cell counts corresponding to the migration and invasion assays for T24 and TSGH 8301 cells treated with U0126 or shERK are shown, demonstrating the same trends as the quantified data presented in Fig. 6.

**Supplementary Fig. 4**. Wnt inhibition abolishes PM_2.5_-induced ERK activation in T24 and TSGH 8301 cells. T24 and TSGH 8301 cells were pretreated with Wnt pathway inhibitors (IWP-2: 0.625 μM and BOX-5: 0.75 μg/ml) for one hour prior to PM_2.5_ exposure at 2.5 μg/ml and 1.25 μg/ml, respectively. Western blot analysis demonstrated that, in the absence of Wnt signaling, PM_2.5_ failed to induce ERK phosphorylation. Total ERK served as the loading control, and representative blots from independent experiments are shown. *, p < 0.05; **, p < 0.01; ***, p < 0.001; ****, p < 0.0001; * represented the comparison with control group; # represented the comparison with PM_2.5_ group.

**Supplementary Fig. 5**. Protein expression levels in the non-canonical Wnt and MAPK pathways after PM_2.5_ exposure. PM: particulate matter; MAPK: mitogen-activated protein kinase. *, p < 0.05; **, p < 0.01.
